# Retention Database for Prediction, Simulation, and Optimization of GC Separations

**DOI:** 10.1021/acsomega.3c01348

**Published:** 2023-05-23

**Authors:** Tillman Brehmer, Benny Duong, Manuela Marquart, Luise Friedemann, Peter J. Faust, Peter Boeker, Matthias Wüst, Jan Leppert

**Affiliations:** † Institute of Nutritional and Food Sciences, Food Chemistry, University of Bonn, Endenicher Allee 11−13, 53115 Bonn, Germany; ‡ Department for Applied Sciences, Hochschule Bonn-Rhein-Sieg, Von-Liebig-Straße 20, 53359 Rheinbach, Germany; § HyperChrom GmbH Germany, Endenicher Allee 11−13, 53115 Bonn, Germany

## Abstract

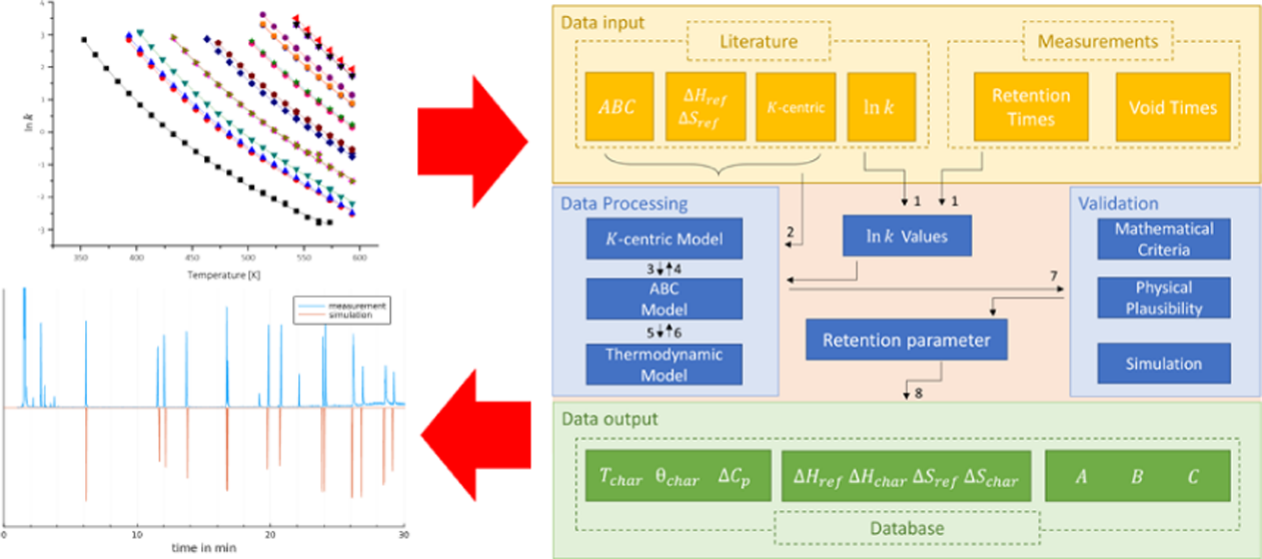

This work presents an open source database with suitable retention parameters for prediction and simulation of GC separations and gives a short introduction to three common retention models. Useful computer simulations play an important role to save resources and time in method development in GC. Thermodynamic retention parameters for the ABC model and the *K*-centric model are determined by isothermal measurements. This standardized procedure of measurements and calculations, presented in this work, have a useful benefit for all chromatographers, analytical chemists, and method developers because it can be used in their own laboratories to simplify the method development. The main benefits as simulations of temperature-programed GC separations are demonstrated and compared to measurements. The observed deviations of predicted retention times are in most cases less than 1%. The database includes more than 900 entries with a large range of compounds such as VOCs, PAHs, FAMEs, PCBs, or allergenic fragrances over 20 different GC columns.

## 1 Introduction

Method developments in gas (liquid) chromatography can often require a lot of time and resources. More efficient, less expensive, and resource-saving perspectives are opened up by the use of appropriate computer simulations to simplify the optimization process and solve separation problems. In method development, even simple retention models and calculations can be very helpful, for example, to estimate elution orders, retention times, or resolution. Retention models and simulations need substance-specific retention parameters, for example, for the model of Clarke and Glew^1^ or the *K*-centric model of Blumberg.^2−4^ Because the determination of those substance-specific and stationary-phase-specific parameters is also elaborate, it is constructive to collect them in databases and share them with the scientific community.

There are other retention databases existing, such as the retention index (RI) database for example of NIST^5^ or the linear solvation energy relationship (LSER) database of UFZ.^6^ These retention data are primarily suitable for prediction of retention phenomena and the distribution in the chromatographic phases. With *K*-centric data, the characteristic temperature may also be suitable for identification. Via simulation, those retention data can also be used for prediction of retention indices similar to the LSER approach.^7^ The retention data presented in this work are temperature-independent and can therefore be used for prediction of temperature programs.^8^ Therefore, compared to the LSER^9^ approach *K*-centric retention data can describe the change of retention factor *k* with the temperature.

This work presents an available open source retention database for three common retention models and gives a short overview for the calculation of the corresponding data. All three retention models describe the temperature dependence of the retention factor with different parameter sets and can be converted into each other. To save the user of the database a conversion, the data for each of the three retention models are presented in the corresponding parameter set, which is very convenient. The benefit of the data, for example, for simulation of GC separations is demonstrated. The standardized procedure of the determination can be useful for every gas chromatographer or analytical chemist to get predictions for their own measurements.

### 1.1 Thermodynamic Retention Model

In gas chromatography, the partition of a solute between the mobile phase (gas) and the stationary phase (liquid) is measured by the distribution coefficient *K*, defined as the ratio of the concentration of the solute in the stationary phase and in the mobile phase. It can be measured by isothermal measurements of the retention factor *k* and the phase ratio β of the column.
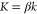
1

The distribution coefficient *K* depends on the temperature *T* and the Gibbs free energy Δ*G* of the evaporation of the solute from the stationary phase.^10^
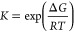
2with *R* being the molar gas constant. The Gibbs free energy Δ*G* can be expressed by enthalpy Δ*H* and entropy Δ*S* changes of the solute from the stationary into the mobile phase as
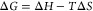
3and therefore
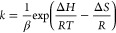
4

Both Δ*H* and Δ*S* depend on the temperature *T* itself. To compensate for this temperature dependency, a third parameter Δ*C*_*p*_ (change of the isobaric molar heat capacity) can be introduced and the enthalpy Δ*H*_ref_ and entropy Δ*S*_ref_ at a reference temperature *T*_ref_ are used. Equations 2 and 3 lead to the classic van’t Hoff model and further to
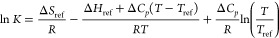
5which can be converted in a 3-parameter model of Clarke and Glew^1,4^ for curve fitting
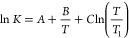
6

It was shown^11^ that using a 3-parameter model results in a better fit of *k* over a wider temperature range than using a 2-parameter model with constant Δ*H* and Δ*S*.

The parameters *A*, *B*, and *C* can be converted to enthalpy Δ*H*_ref_ and entropy Δ*S*_ref_ for a chosen reference temperature *T*_ref_ and the change of the isobaric molar heat capacity Δ*C*_*p*_.
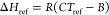
7
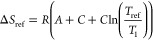
8
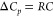
9

It seems reasonable to set up a model that normalizes its reference variables to a certain temperature. In adsorption phenomena, especially in chromatography, the distribution of an analyte depends to a large extent on the temperature conditions but not on the same temperature for each analyte. Choosing one reference temperature *T*_ref_ for all analytes leads to physically meaningless conditions for substances with extreme retention, such as highly volatile compounds or low volatile substances like triglycerides. For chromatography, it is more appropriate to normalize the model to the same distribution of the analyte over the stationary phase, expressed by the distribution coefficient *K*.^4^

A fully equivalent model to describe the distribution of a solute between stationary and mobile phases in a 3-parameter model is the distribution-centric 3-parameter model of Blumberg,^4^ the short *K*-centric model. In this model, the retention factor *k* of a solute in a GC system is defined by three parameters:

- *T*_char_ characteristic temperature
- θ_char_ characteristic thermal constant
- Δ*C*_*p*_ change of the isobaric molar heat capacity (eq 7)

and the equation
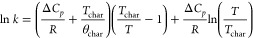
10

These parameters, especially *T*_char_ and θ_char_, have a direct chromatographic meaningful interpretation. The characteristic temperature *T*_char_ is the temperature, where ln *k* = 0 and *k* = 1.^4^ At this temperature, the amount of the solute is evenly distributed between stationary and mobile phases. The characteristic thermal constant is the inverse declining slope of the function ln *k*(*T*) at *T* = *T*_char_. Therefore, an increase of the temperature around *T*_char_ by θ_char_ reduces *k* by a factor of *e* ≈ 2.72. The interpretation of Δ*C*_*p*_ is not straightforward, but it generally defines the deviation of *k* from a 2-parameter model for temperature significantly lower/higher than *T*_char_.

The parameters *T*_char_, θ_char_, and Δ*C*_*p*_ are specific for the phase ratio β_0_ used to determine these parameters. Using a column with the same stationary phase but different phase ratio β_1_ requires a correction factor for the retention factor calculated from eq 10.
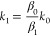
11

The retention factor *k* can be determined using the retention time from the chromatogram at the known void time *t*_M_ of the GC column, which is the time the carrier gas or a substance with no retention requires to pass the column.
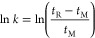
12

The void time *t*_M_ can be measured by detection of a non-interacting gas, for example, methane or air. For wall-coated cylindrical GC columns with length *L*, internal diameter *d*, and temperature *T*, *t*_M_ can also be determined with
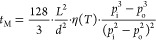
13where *p*_i_ is the pressure at the inlet of the column, *p*_o_ at the column outlet, and η is the viscosity of the carrier gas.^10^

## 2 Materials and Methods

### 2.1 Chemicals

To create the database, 260 substances were measured, such as homologous alkanes, alcohols, ketones, phenones, BTEXs, halogen-phenols, and others. Relevant substances for the analytic in food and cosmetics were also measured, for example, 37 FAMEs, 58 allergenic fragrances, 16 EPA-PAHs, 6 PCBs, 6 triglycerides, and other volatile compounds. All used standard substances were purchased by Sigma-Aldrich with a purity of higher than 99.9%. Therefore, dilutions of the compounds were used to determine retention parameters of these substances and to measure chromatograms with different temperature programs.

### 2.2 Columns

Measurements for determination of the retention parameters were performed on different GC separation columns: 30 m × 0.25 mm × 0.25 μm Rxi17SilMS (75% phenyl–25% methylpolysiloxane, Restek. USA), 30 m × 0.25 mm × 0.25 μm Rxi5SilMS (75% phenyl–25% methylpolysiloxane, Restek. USA), 30 m × 0.25 mm × 0.5 μm Rxi5SilMS, and 10 m × 0.1 mm × 0.1 μm ZB-PAH-CT (proprietary stationary phase, Phenomenex, USA). Void times were measured with injections of air and detection of the oxygen signal in the TOF-MS. The *L*/*d* ratios of the columns were determined from void time measurements by using eq 13 and are shown in Table 1.

**Table 1:** Determined *L*/*d* Ratios for the Investigated Separation Columns

stationary phase	*d* [mm]	*d*_f_ [μm]	*L*/*d*	*L* [m]
Rxi17SilMS	0.25	0.25	120,889.6 ± 170.4	30.222 ± 0.043
Rxi5SilMS	0.25	0.25	121,606.8 ± 1475.7	30.40 ± 0.37
Rxi5SilMS	0.25	0.5	119,084.0 ± 1276.0	29.77 ± 0.32
ZB-PAH-CT	0.1	0.1	102,300.0 ± 4700.0	10.23 ± 0.47

### 2.3 Instrumentals

A HP 6890 series GC system from Hewlett Packard/Agilent with split/splitless injector (300 °C, 1:100 split ratio) coupled with a BenchTOF-dx time-of-flight mass spectrometer from Markes, UK, was used. The allergen fragrances on the Rxi17SilMS were measured using an internal flame ionization detector of the GC (HP), with void time measurements using methane. Carrier gas was helium with purity of 99.9%. A PAL RSI Chronect Robotic autosampler (CTC Analytics AG, Switzerland) was used for injection of 1 μL of each sample. Isothermal measurements were made in the range from 60 to 300 °C with 10 °C increments and a constant flow of 1 mL/min of the carrier gas.

To validate the parameters, temperature-programed measurements were performed on the HP 6890 GC and a flow field gradient GC (FF-TG-GC)^12^ (HyperChrom SA, Luxembourg). The measured chromatograms were compared to simulated data.

### 2.4 Literature Data

13 data sets with retention parameters were found in the literature. Table 2 gives an overview about the size of the data sets, the number of compounds and columns that are included, and the reference of the literature.

**Table 2:** Data sets with Retention Data Found in the Literature That are Included in the Database

data set	size of data set	number of compounds	number of columns	references
1	88	88	1	(13)
2	47	45	1	(14)
3	5	5	1	(3)
4	7	7	1	(15)
5	51	17	3	(11)
6	22	22	1	(2)
7	76	12	3	(16)
8	6	6	1	(17)
9	25	11	3	(18)
10	11	11	1	(19)
11	25	19	1	(20)
12	34	16	2	(21)
13	135	19	8	(22)

### 2.5 Software

For calculation of void times and ln *k* values, MS Office Professional Plus 2019 Excel was used. All other calculations were performed in a Pluto notebook^23^ using the programming language Julia.^24^ The notebook is available in the project “RetentionData” via GitHub.^25^ For robust fitting and outlier detection, the package RAFF.jl was used.^26^ For linear and multivariate fits, the package LsqFit.jl was used.^27,28^ Simulation of GC separations and chromatograms were performed with the open source software GasChromatographySimulator.jl.^29^ Detailed information to the simulation can be found elsewhere.^2^

## 3 Creation of the Database

### 3.1 Calculations and Processing Steps

**Figure 1:**
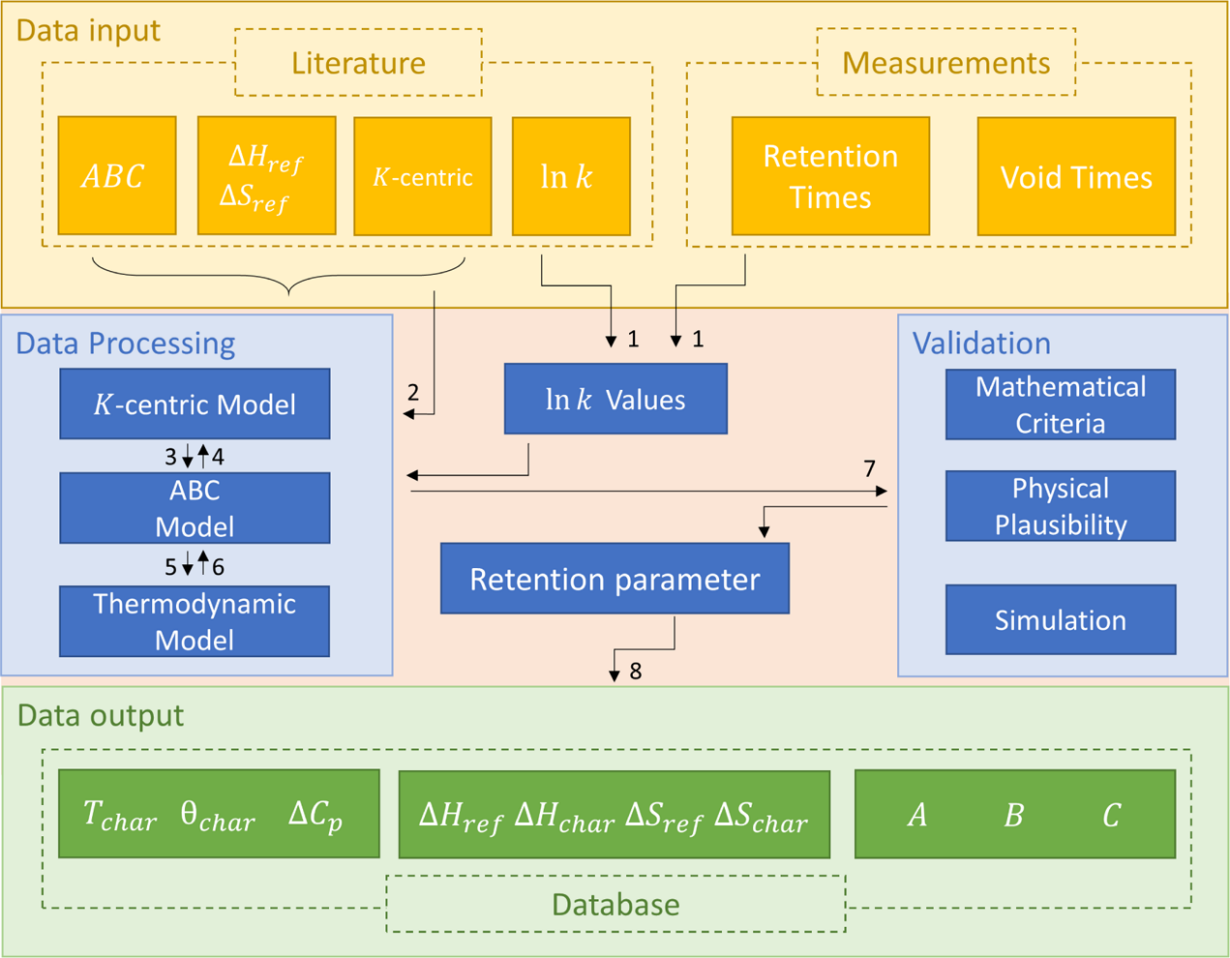
Schematic overview of the main tasks for calculation and converting of the retention parameters and creation of the database.

A schematic overview of the calculation and processing steps is given in Figure 1.

*K*-centric parameters of each compound were determined by fitting the ln *k* values, calculated by eq 12, against the temperature of the investigated temperature range by using the *K*-centric model by Blumberg (eq 10) (see Figure 1 no. 1).

*K*-centric parameters were converted into the ABC parameters using eq 14 (see Figure 1 no. 3) with knowledge of nominal β.^4^
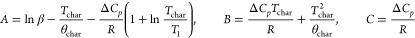
14

Enthalpy Δ*H*_ref_ and entropy Δ*S*_ref_ were determined from the ABC parameters by using eq 7 and 8, respectively, with a reference temperature of 90 °C (Figure 1 no. 5). 90 °C for *T*_ref_ was chosen because other the literature data are determined at these reference temperatures. With *T*_ref_ = *T*_char_, the *K*-centric equivalents Δ*H*_char_ and Δ*S*_char_, enthalpy, and entropy at the solute specific characteristic temperature were determined, which are more meaningful for chromatography.^4^

Data from the literature were converted into *K*-centric parameters by using the following steps (Figure 1 no. 2).

ABC parameters can be converted to *K*-centric data by using eqs 15 and 16^4^ (Figure 1 no. 4).^4^
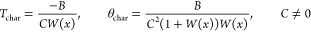
15with
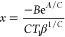
16where *T*_1_ = 1 K and *W*(*x*) is the Lambert *W* function (also known as product log function). Per definition, the argument *x* has to be larger than −1/*e*. The Lambert *W* function has two branches *W*_0_ and *W*_–1_, as shown in Supporting Information, Figure S1. All data so far, show that only the branch *W*_–1_ is used; therefore, the value of *x*, eq 16, has to be between −1/*e* and 0.

With knowledge of the reference temperature *T*_ref_, thermodynamic data as Δ*S*_ref_ and Δ*H*_ref_ can be converted into ABC parameters^4^ (Figure 1 no. 6). As shown above, they can be converted into *K*-centric data (Figure 1 no. 4).

### 3.2 Validation and Quality Control

The calculated values have to be validated (Figure 1 no. 7). For acceptance of the compound data the following criteria are defined:

(a) The data set includes three data points as minimum for non-linear multivariate fit, ideally four data points or more. As a recommendation, the data should contain points around ln *k* = 0 to achieve accurate fitting results.
(b) ln *k* values range between −2.0 and 3.5, too high ln *k* values are associated with too broad peaks, increased signal-to-noise, and inaccurate retention times. Since low ln *k* values often result in analyte peaks merging into the solvent peak, retention does not only depend on the stationary phase.
(c) 0 < θ_char_ < 100, a negative θ_char_ cannot be accepted because it would mean that a temperature increase leads to higher retention times than to lower. Based on available data, the parameter θ_char_ tends to be lower than 100 °C, in most cases around 30 °C.^10^
(d) *T*_char_ > −273.15 °C, a value of *T*_char_ below the absolute zero is not possible.
(e) *C* > 0, negative *C* shows a lower bending of the fit curve, the curve becomes more linear and causes also to the wrong branch of the Lambert *W* function (*W*_0_).
(f) *A* < 0, based on available data the parameter *A* tends to be negative.
(g) *W*(*x*) < −1 and −1/*e* < *x* < 0, data are inacceptable if the value of the argument *x* of the Lambert *W* function gets lower than −1/*e* or *W*(*x*) > −1. Available data shows a value of *W*(*x*) lower than −1 and is on the *W*_–1_ branch, therefore −1/*e* < *x* < 0.

Data that failed one of the criteria will be flagged in the database. The reason of the failure will be documented.

To create the final database after validation as shown in Figure 1 no. 8, the parameters of each compound related to the stationary phase are collected in a table. For many substances, a substance category is added, for example “*n*-alkanes” for homologous series of alkanes, “FAMEs” for fatty acid methyl esters (FAMEs), or “Grob”, if the substance is part of the Grob mix for evaluation of GC columns. The structure of the final table is shown in Table 3.

**Table 3:** Structure of the Retention Database and Determined Values of the Retention Parameters of a Selection of Allergenic Fragrances, Triglycerides, PCBs, and PAHs^a^

name	CAS	phase	φ_0_	*A*	error_A_	*B*	error_B_	*C*	error_C_	Δ*H*_ref_	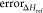	Δ*S*_ref_	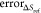	*T*_ref_
cinnamaldehyde	104-55-2	Rxi17SilMS	0.001	–82.062	0.65699	10,505	41.611	10.503	0.092476	–55,627	74.286	–80.181	0.17972	90
farnesol A	4602-84-0	Rxi17SilMS	0.001	–108.94	5.955	13,698	402.29	13.933	0.82925	–71,825	876.98	–107.01	2.0583	90
farnesol B	4602-84-0	Rxi17SilMS	0.001	–143.29	4.9454	15,819	337.2	18.806	0.68763	–74,741	757.59	–113.34	1.7679	90
geraniol	106-24-1	Rxi17SilMS	0.001	–88.825	2.3832	10,625	143.78	11.451	0.33807	–53,770	199.92	–82.087	0.49881	90
glyceryl tridecanoate	621-71-6	Rxi17SilMS	0.001	–394.74	65.672	41,657	5590.7	51.841	8.8180	–189,820	20186	–310.16	41.164	90
glyceryl trihexanoate	621-70-5	Rxi17SilMS	0.001	–188.84	20.960	22,064	1588.2	24.456	2.8654	–109,610	4636.7	–168.13	10.181	90
glyceryl trilaurate	538-24-9	Rxi17SilMS	0.001	–543.67	127.33	55,893	11394	71.568	16.969	–248,630	44256	–417.58	87.316	90
glyceryl trimyristin	555-45-3	Rxi17SilMS	0.001	–655.14	198.66	66,369	27435	86.428	25.872	–290,860	181970	–492.57	265.34	90
glyceryl trioctanoate	538-23-8	Rxi17SilMS	0.001	–211.38	27.344	25,948	2197.4	27.112	3.7033	–133,880	7159.8	–203.29	15.197	90
glyceryl tripalmitin	555-44-2	Rxi17SilMS	0.001	–493.02	661.79	53,703	529250	64.545	23.508	–251,620	4398400	–399.00	5293.9	90
iso E super A	54464-57-2	Rxi17SilMS	0.001	–94.965	2.4567	12,303	167.07	12.154	0.34177	–65,595	375.22	–92.836	0.87513	90
iso E super B	54464-57-2	Rxi17SilMS	0.001	–96.736	3.5892	12,420	243.95	12.407	0.49917	–65,801	531.2	–93.049	1.2494	90
iso E super C	54464-57-2	Rxi17SilMS	0.001	–96.021	5.5070	12,334	376.07	12.331	0.76535	–65,315	833.99	–91.485	1.9543	90
iso E super D	54464-57-2	Rxi17SilMS	0.001	–101.18	5.3862	12,802	368.57	13.014	0.74834	–67,148	823.79	–95.219	1.9272	90
limonene	138-86-3	Rxi17SilMS	0.001	–75.098	4.3818	83,93.5	240.6	9.8499	0.63146	–40,046	205.41	–59.733	0.54245	90
linalool	78-70-6	Rxi17SilMS	0.001	–83.176	2.6778	95,08.6	151.66	10.803	0.38383	–46,441	140.04	–72.290	0.36234	90
PCB 101	37680-73-2	Rxi5SilMS	0.002	–111.16	0.85969	14,804	66.924	14.179	0.11708	–80,277	209.05	–111.41	0.44911	90
PCB 138	35065-28-2	Rxi5SilMS	0.002	–107.39	2.4739	15,187	200.61	13.561	0.33499	–85,325	685.92	–115.53	1.4259	90
PCB 153	35065-27-1	Rxi5SilMS	0.002	–106.02	3.2438	14,985	260.39	13.377	0.43993	–84,199	874.59	–114.60	1.8301	90
PCB 180	35065-29-3	Rxi5SilMS	0.002	–95.041	5.9211	14,747	489.4	11.783	0.79956	–87,035	1731	–114.72	3.5510	90
PCB 28	7012-37-5	Rxi5SilMS	0.002	–101.55	2.2747	13,300	169.51	13.001	0.31176	–71,330	474.46	–98.992	1.0547	90
PCB 52	35693-99-3	Rxi5SilMS	0.002	–108.61	2.3285	14,057	175.54	13.925	0.31859	–74,832	506.49	–104.77	1.1160	90
benz[*a*]anthracene	56-55-3	ZB-PAH-CT	0.001	–17.543	48.568	9795.2	3816.2	0.92277	6.6114	–78,656	12497	–92.960	26.368	90
benzo[*g*,*h*,*i*]perylene	191-24-2	ZB-PAH-CT	0.001	–15.308	13.044	10,841	1125.3	0.57344	1.7504	–88,404	4257.1	–94.403	8.4867	90
dibenzo[*a*,*h*]anthracene	53-70-3	ZB-PAH-CT	0.001	–93.497	26.461	16,475	2300.0	11.327	3.5481	–10,2780	8853.9	–128.05	17.501	90
indeno[1,2,3-*cd*]pyrene	193-39-5	ZB-PAH-CT	0.001	–55.231	39.696	13,583	3436.5	6.0911	5.3263	–94,547	13175	–110.03	26.091	90

name	CAS	phase	φ_0_	*T*_*c*har_	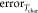	θ_char_	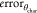	Δ*C*_*p*_	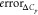	v*H*_char_	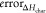	Δ*S*_char_	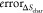
cinnamaldehyde	104-55-2	Rxi17SilMS	0.001	174.33	0.012622	34.497	0.025762	87.329	0.76889	–48262	36.144	–61.944	0.0806
farnesol A	4602-84-0	Rxi17SilMS	0.001	210.18	0.075096	33.545	0.16568	115.85	6.8948	–57,903	286.56	–73.892	0.59201
farnesol B	4602-84-0	Rxi17SilMS	0.001	215.35	0.069363	35.982	0.15936	156.36	5.7173	–55,141	244.72	–66.971	0.50020
geraniol	106-24-1	Rxi17SilMS	0.001	150.5	0.032493	31.082	0.067725	95.209	2.8109	–48,010	104.87	–67.417	0.24708
glyceryl tridecanoate	621-71-6	Rxi17SilMS	0.001	357.50	1.20	44.375	2.8054	431.03	73.317	–74,520	4719.7	–72.255	7.4737
glyceryl trihexanoate	621-70-5	Rxi17SilMS	0.001	279.28	0.36766	35.679	0.53451	203.34	23.824	–71,117	1069.6	–82.828	1.9305
glyceryl trilaurate	538-24-9	Rxi17SilMS	0.001	393.69	3.3938	54.435	8.6086	595.05	141.09	–67,920	10764	–55.946	16.116
glyceryl trimyristin	555-45-3	Rxi17SilMS	0.001	471.37	130.28	274.27	2095.4	718.60	215.11	–16,804	128520	23.337	172.49
glyceryl trioctanoate	538-23-8	Rxi17SilMS	0.001	318.75	0.27448	35.386	0.54949	225.42	30.791	–82,318	1280.5	–93.167	2.1606
glyceryl tripalmitin	555-44-2	Rxi17SilMS	0.001	556.97	5727.5	5634.7	17350000	536.66	195.45	–10,16.8	3131000	44.683	3771.7
iso E super A	54464-57-2	Rxi17SilMS	0.001	213.41	0.043614	37.053	0.092552	101.05	2.8417	–53,123	133.03	–63.273	0.27289
iso E super B	54464-57-2	Rxi17SilMS	0.001	214.72	0.025811	37.385	0.084073	103.16	4.1503	–52,935	119.17	–62.594	0.24407
iso E super C	54464-57-2	Rxi17SilMS	0.001	217.20	0.046149	38.245	0.14648	102.52	6.3635	–52,274	200.45	–60.696	0.40842
iso E super D	54464-57-2	Rxi17SilMS	0.001	218.27	0.047197	37.694	0.14396	108.20	6.222	–53,269	203.7	–62.489	0.41412
limonene	138-86-3	Rxi17SilMS	0.001	106.20	0.05929	30.903	0.1486	81.897	5.2502	–38,719	186.58	–56.158	0.49107
linalool	78-70-6	Rxi17SilMS	0.001	120.72	0.029744	29.529	0.067299	89.817	3.1914	–43,682	99.774	–64.996	0.25290
PCB 101	37680-73-2	Rxi5SilMS	0.002	294.81	0.023455	47.780	0.053172	117.89	0.97344	–56,132	62.638	–58.687	0.11006
PCB 138	35065-28-2	Rxi5SilMS	0.002	318.7	0.12712	48.915	0.20645	112.75	2.7853	–59,539	252.59	–60.455	0.42514
PCB 153	35065-27-1	Rxi5SilMS	0.002	312.05	0.16064	47.854	0.25766	111.22	3.6578	–59,502	322.03	–61.532	0.54816
PCB 180	35065-29-3	Rxi5SilMS	0.002	330.90	0.33185	47.826	0.48932	97.972	6.6479	–63,433	652.73	–64.868	1.0760
PCB 28	7012-37-5	Rxi5SilMS	0.002	269.33	0.052128	47.105	0.085008	108.10	2.5921	–51,944	94.272	–55.609	0.17305
PCB 52	35693-99-3	Rxi5SilMS	0.002	276.10	0.050548	47.072	0.10184	115.78	2.6489	–53,285	115.7	–56.871	0.21008
benz[*a*]anthracene	56-55-3	ZB-PAH-CT	0.001	296.00	2.5171	34.944	2.3759	7.6724	54.97	–77,075	5284.5	–89.513	9.2267
benzo[*g*,*h*,*i*]perylene	191-24-2	ZB-PAH-CT	0.001	359.69	0.82067	38.222	0.71626	4.7678	14.553	–87,118	1648.1	–91.755	2.5859
dibenzo[*a*,*h*]anthracene	53-70-3	ZB-PAH-CT	0.001	362.95	2.0216	43.648	2.0490	94.176	29.501	–77,076	3651.2	–75.261	5,7012
indeno[1,2,3-cd]pyrene	193-39-5	ZB-PAH-CT	0.001	359.73	3.0879	41.172	2.7876	50.644	44.285	–80,886	5533.2	–81.899	8.6760

name	CAS	phase	φ_0_	*N*	*R*^2^	χ^2^	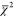	source	flag	category 1	category 2
cinnamaldehyde	104-55-2	Rxi17SilMS	0.001	8	1	6.5340 × 10^–7^	1.3068 × 10^–7^	this work		aldehyde	allergenic fragrances
farnesol A	4602-84-0	Rxi17SilMS	0.001	8	0.99999	3.6088 × 10^–5^	7.2175 × 10^–6^	this work		allergenic fragrances	
farnesol B	4602-84-0	Rxi17SilMS	0.001	9	0.99999	5.2339 × 10^–5^	8.7231 × 10^–6^	this work		allergenic fragrances	
geraniol	106-24-1	Rxi17SilMS	0.001	9	1	2.2404 × 10^–5^	3.7340 × 10^–6^	this work		terpene	allergenic fragrances
glyceryl tridecanoate	621-71-6	Rxi17SilMS	0.001	24	0.99636	8.3195 × 10^–2^	3.9617 × 10^–3^	this work		triglyceride	
glyceryl trihexanoate	621-70-5	Rxi17SilMS	0.001	14	0.99968	9.3626 × 10^–3^	8.5115 × 10^–4^	this work		triglyceride	
glyceryl trilaurate	538-24-9	Rxi17SilMS	0.001	13	0.99883	3.9493 × 10^–3^	3.9493 × 10^–4^	this work		triglyceride	
glyceryl trimyristin	555-45-3	Rxi17SilMS	0.001	13	0.99916	9.1803 × 10^–3^	9.1803 × 10^–4^	this work	θ_char_ > 100 °C	triglyceride	
glyceryl trioctanoate	538-23-8	Rxi17SilMS	0.001	20	0.99958	1.4787 × 10^–2^	8.6984 × 10^–4^	this work		triglyceride	
glyceryl tripalmitin	555-44-2	Rxi17SilMS	0.001	9	0.99897	3.4491 × 10^–3^	5.7485 × 10^–4^	this work	θ_char_ > 100 °C	triglyceride	
iso E super A	54464-57-2	Rxi17SilMS	0.001	10	1	2.7114 × 10^–5^	3.8735 × 10^–6^	this work		allergenic fragrances	
iso E super B	54464-57-2	Rxi17SilMS	0.001	7	1	4.5734 × 10^–6^	1.1434 × 10^–6^	this work		allergenic fragrances	
iso E super C	54464-57-2	Rxi17SilMS	0.001	7	1	1.0752 × 10^–5^	2.6879 × 10^–6^	this work		allergenic fragrances	
iso E super D	54464-57-2	Rxi17SilMS	0.001	7	1	1.0279 × 10^–5^	2.5697 × 10^–6^	this work		allergenic fragrances	
limonene	138-86-3	Rxi17SilMS	0.001	5	1	4.0222 × 10^–6^	2.0111 × 10^–6^	this work		terpene	allergenic fragrances
linalool	78-70-6	Rxi17SilMS	0.001	8	1	1.7043 × 10^–5^	3.4086 × 10^–6^	this work		allergenic fragrances	terpene
PCB 101	37680-73-2	Rxi5SilMS	0.002	10	1	5.0424 × 10^–6^	7.2034 × 10^–7^	this work		PCB	
PCB 138	35065-28-2	Rxi5SilMS	0.002	11	1	4.0017 × 10^–5^	5.0021 × 10^–6^	this work		PCB	
PCB 153	35065-27-1	Rxi5SilMS	0.002	10	1	4.1581 × 10^–5^	5.9401 × 10^–6^	this work		PCB	
PCB 180	35065-29-3	Rxi5SilMS	0.002	9	0.99999	8.5642 × 10^–5^	1.4274 × 10^–5^	this work		PCB	
PCB 28	7012-37-5	Rxi5SilMS	0.002	11	0.99999	5.4783 × 10^–5^	6.8479 × 10^–6^	this work		PCB	
PCB 52	35693-99-3	Rxi5SilMS	0.002	12	0.99999	6.5045 × 10^–5^	7.2273 × 10^–6^	this work		PCB	
benz[*a*]anthracene	56-55-3	ZB-PAH-CT	0.001	8	0.99975	1.5696 × 10^–3^	3.1392 × 10^–4^	this work		PAH	
benzo[*g*,*h*,*i*]perylene	191-24-2	ZB-PAH-CT	0.001	8	0.99999	7.7010 × 10^–5^	1.5402 × 10^–5^	this work		PAH	
dibenzo[*a*,*h*]anthracene	53-70-3	ZB-PAH-CT	0.001	8	0.99994	3.1643 × 10^–4^	6.3287 × 10^–5^	this work		PAH	
indeno[1,2,3-*cd*]pyrene	193-39-5	ZB-PAH-CT	0.001	15	0.9996	4.1942 × 10^–3^	3.4952 × 10^–4^	this work		PAH	

a For each entry, *N* gives the number of measurement points which were used for the fit. φ_0_ is the dimensionless film thickness with φ_0_ = 1/4β.

## 4 Results and Discussion

### 4.1 Determined Parameters

**Figure 2:**
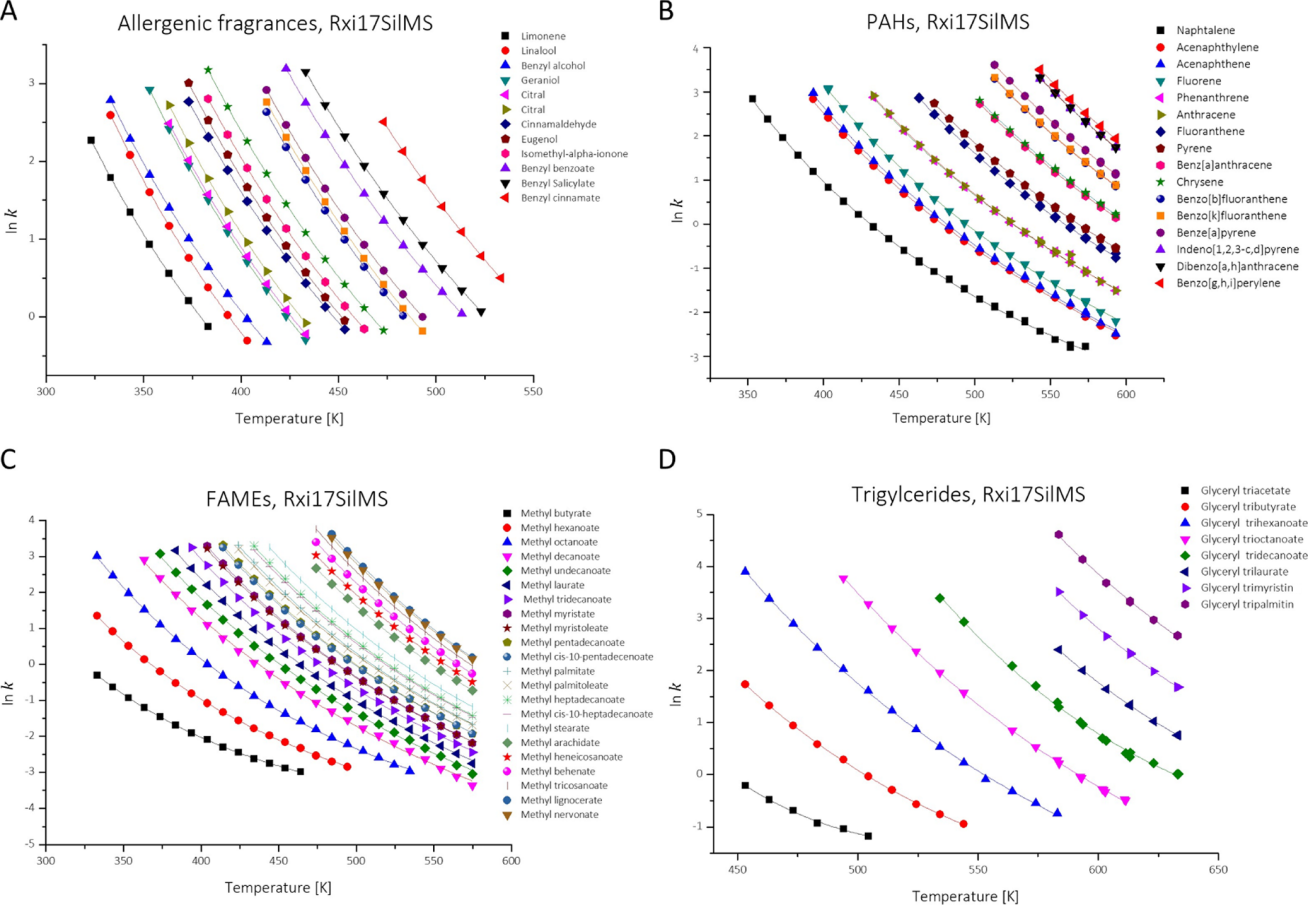
Determined ln *k* values over *T* with fits of the *K*-centric model for each substance for a selection of allergen fragrances (A), EPA-PAHs (B), FAMEs (C), and triglycerides (D) on Rxi17SilMS (β = 250) as the stationary phase.

The determined retention factors from isothermal measurements are plotted against the isothermal temperature. The detailed ln *k* values for each compound can be found in the GitHub project.^25^ The internet link to the data is available in the Supporting Information. The plots and fits as ln *k* over *T* for allergenic compounds, 16 EPA-PAH, FAMEs, and triglycerides on the Rxi17SilMS are shown in Figure 2. The determined retention parameters for the thermodynamic model, the ABC model and the *K*-centric model are shown in the Supporting Information. A selection is shown in Table 3. The value of *N* gives the number of measurements for the fit of each compound.

**Figure 3:**
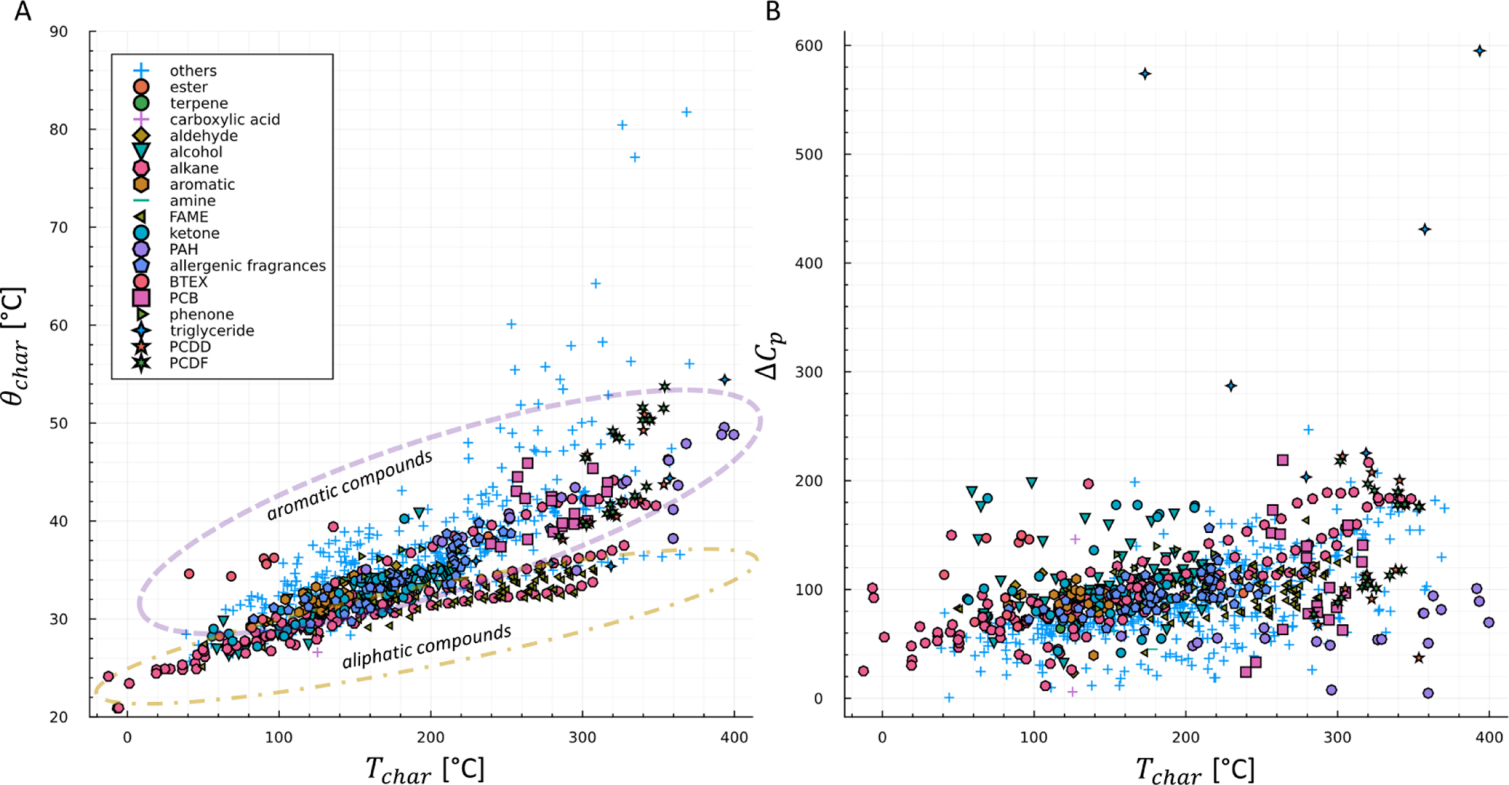
Relationships between *K*-centric parameters and influence of substance category. 2D projection from the 3D parameter space for *T*_char_ against θ_char_ (A) and Δ*C*_*p*_ against *T*_char_ (B).

Figure 3 shows the relationship between the characteristic temperature *T*_char_ and the characteristic thermal constant θ_char_ and to Δ*C*_*p*_. The general relationship is consistent with observations of Blumberg.^10^ A strong influence of different phase ratios on the correlation of θ_char_ on *T*_char_, as described in ref (8) could not be observed in this data. Interactive 3D figures of the *K*-centric and the ABC parameters can be found in Supporting Information, Figures S3 and S4. The ABC data show a nearly straight line in the parameter space. In the parameter space of all three *K*-centric parameters, a general trend can be estimated, whereas some compounds from comparable substance classes show characteristic regions in the space, Figure 3. Aliphatic compounds such as *n*-alkanes, *n*-alcohols, or FAMEs lie in other regions than aromatic compounds such as PAHs, PCBs, or dioxins but even high volatiles like BETXs. The region of the triglycerides is close to FAMEs. Glyceryl trimyristin and glyceryl tripalmitin did not pass the validation because their arguments *x* of the Lambert *W* function are *x* < −1/*e*. A problem during the determination are data measured at high temperature far away from ln *k* = 0, if the parameters, especially *T*_char_, are determined as extrapolation with high standard errors. This can be observed for triglycerides but for some PAHs as well.

**Figure 4:**
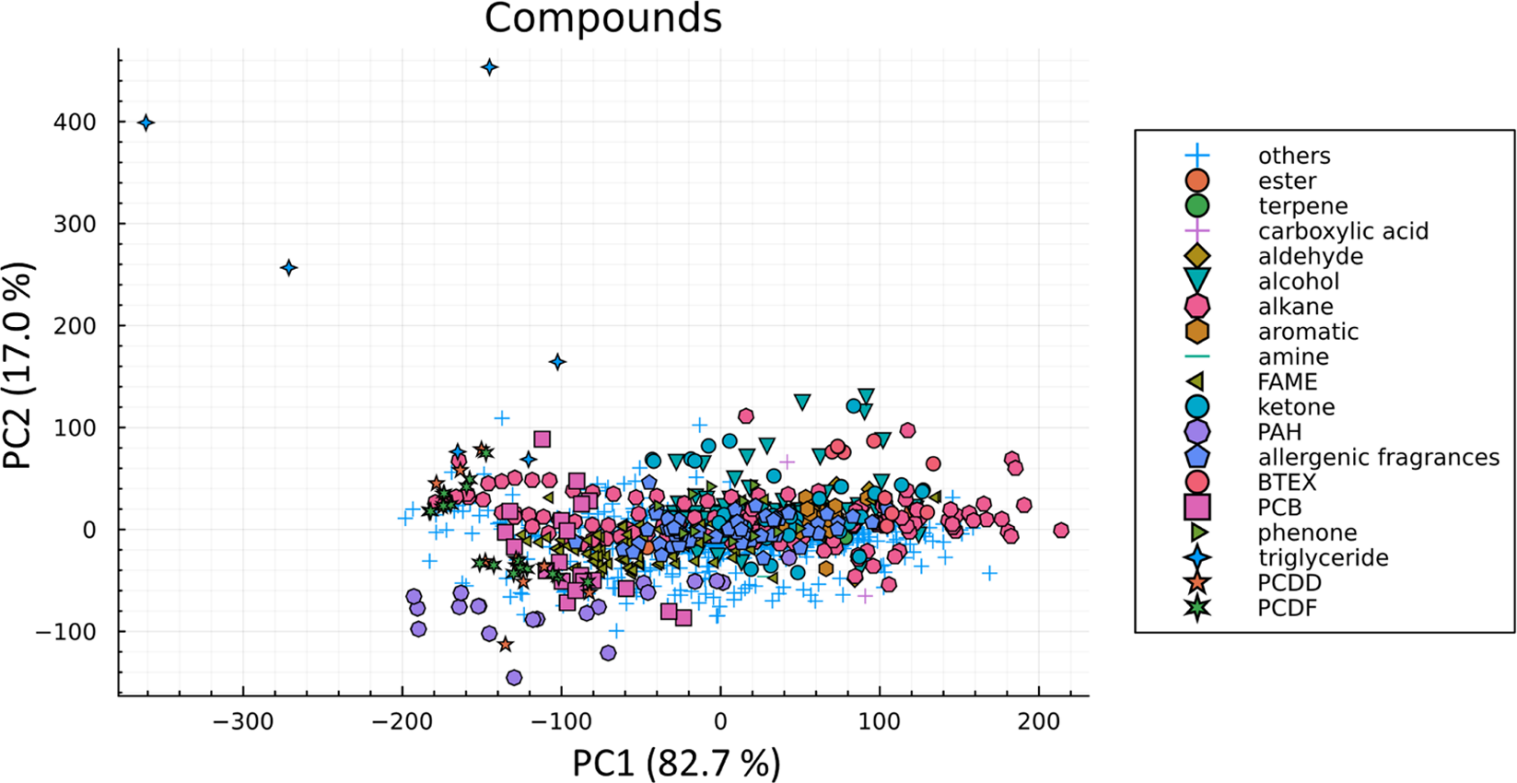
PCA for all three *K*-centric parameters of different compound categories. PC1 explains 82.7% of the data and variance explained = 99.6562.

A principal compound analysis (PCA) provides a model that can describe the relationships between the *K*-centric parameters, Figure 4. PCA of the ABC parameters reduces the data to one principal compound (variance explained = 99.9985%), which is close to the approximately linear trend that could be observed. These PCA models can also be used for further validation of new data and exclusion of data from the database.

### 4.2 Results of the Validation Process

**Figure 5:**
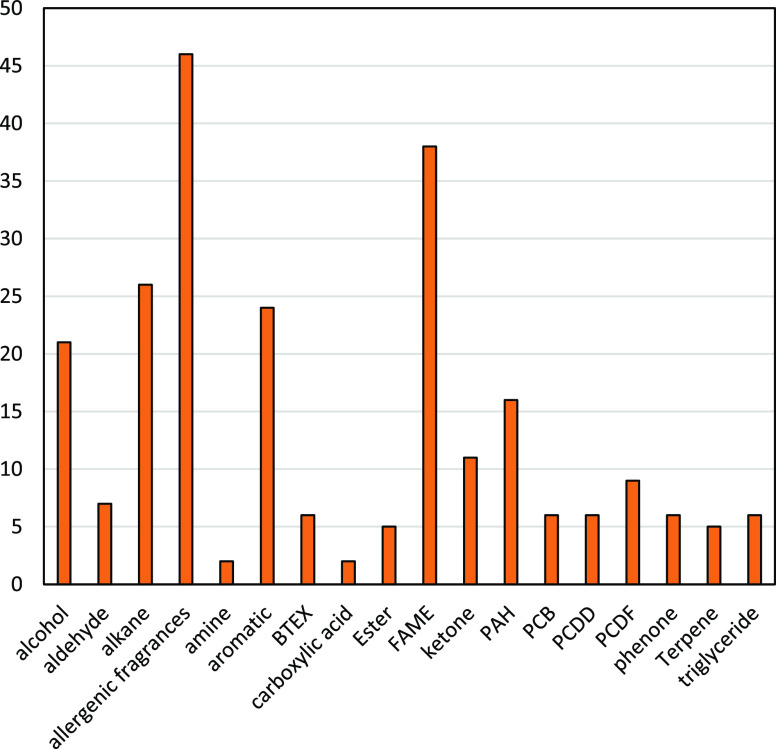
Distribution of different substance categories included in the database (absolute values, substances).

Table 4 shows the final data sets after the validation process. The total size of the database was reduced from 1031 to 967 listings. It is notable, that all of the compounds found by Stevenson et al., did not pass the validation.^20^ This data, obtained by temperature-programed rather than isothermal measurements, show nearly linear ln *k* over *T* curves, so that the Lambert *W* criteria could not be accepted. A similar trend is observed for some of the PAHs measured on the ZB-PAH-Column, which also show very linear curves in the investigated conditions. Figure 5 shows the primary substance categories and the number of compounds in the final database. To review the quality of the determined data, in the next step randomized GC measurements were performed and compared to simulated chromatograms.

**Table 4:** Data sets After the Validation Process Including the Literature Data and Own Determined Data

data set	size of data set before validation	size of data after validation	number of compounds	number of columns	references
1	88	88	88	1	(13)
2	47	47	45	1	(14)
3	5	5	5	1	(3)
4	7	7	7	1	(15)
5	51	51	17	3	(11)
6	22	22	22	1	(2)
7	76	76	12	3	(16)
8	6	6	6	1	(17)
9	25	25	11	3	(18)
10	11	11	11	1	(19)
11	25	0	0	0	(20)
12	34	29	15	2	(21)
13	135	117	19	8	(22)
14	32	22	16	2	this work
15	85	85	70	1	this work
16	355	351	128	3	this work
Total	1031	967	289	20	

### 4.3 Benefit of the Data

**Figure 6:**
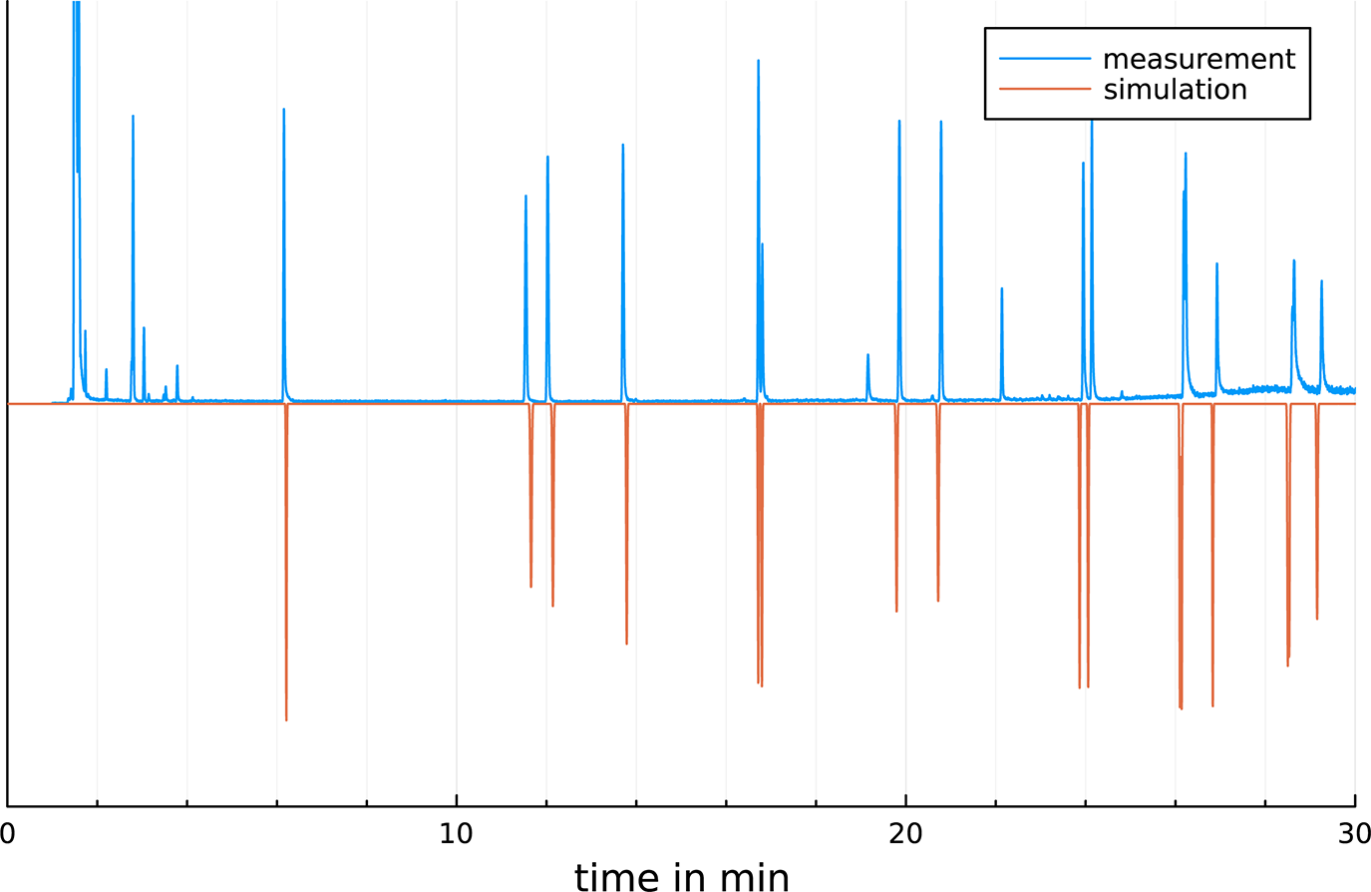
Measured and simulated chromatogram of a temperature-programed GC separation of 16 polycyclic aromatic hydrocarbons (EPA-PAH) on a Rxi17SilMs. GC conditions: *T*_init_ = 70 °C; first ramp: 20 °C/min, *T*_1_ = 150 °C, hold time = 5 min; second ramp: 12 °C/min, *T*_2_ = 250 °C, hold time = 2 min; third ramp: 15 °C/min, *T*_end_ = 360 °C, hold time = 5 min, rmse = 0.1425 min.

**Figure 7:**
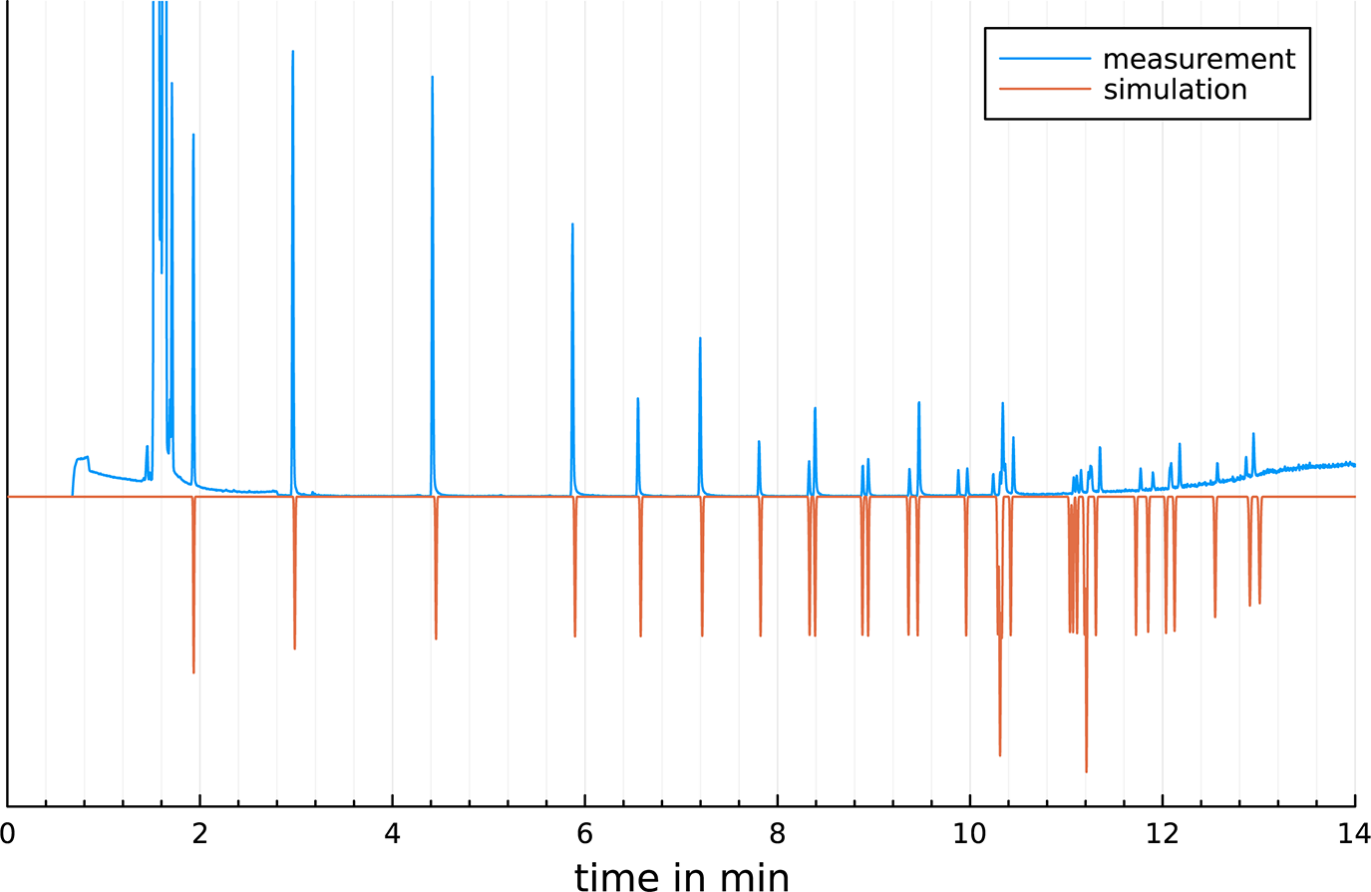
Measured and simulated chromatogram of a temperature-programed GC separation of FAMEs on a Rxi5SilMs. GC conditions: *T*_init_ = 60 °C, first ramp: 20 °C/min, *T*_end_ = 300 °C, rmse = 0.03532 min.

**Figure 8:**
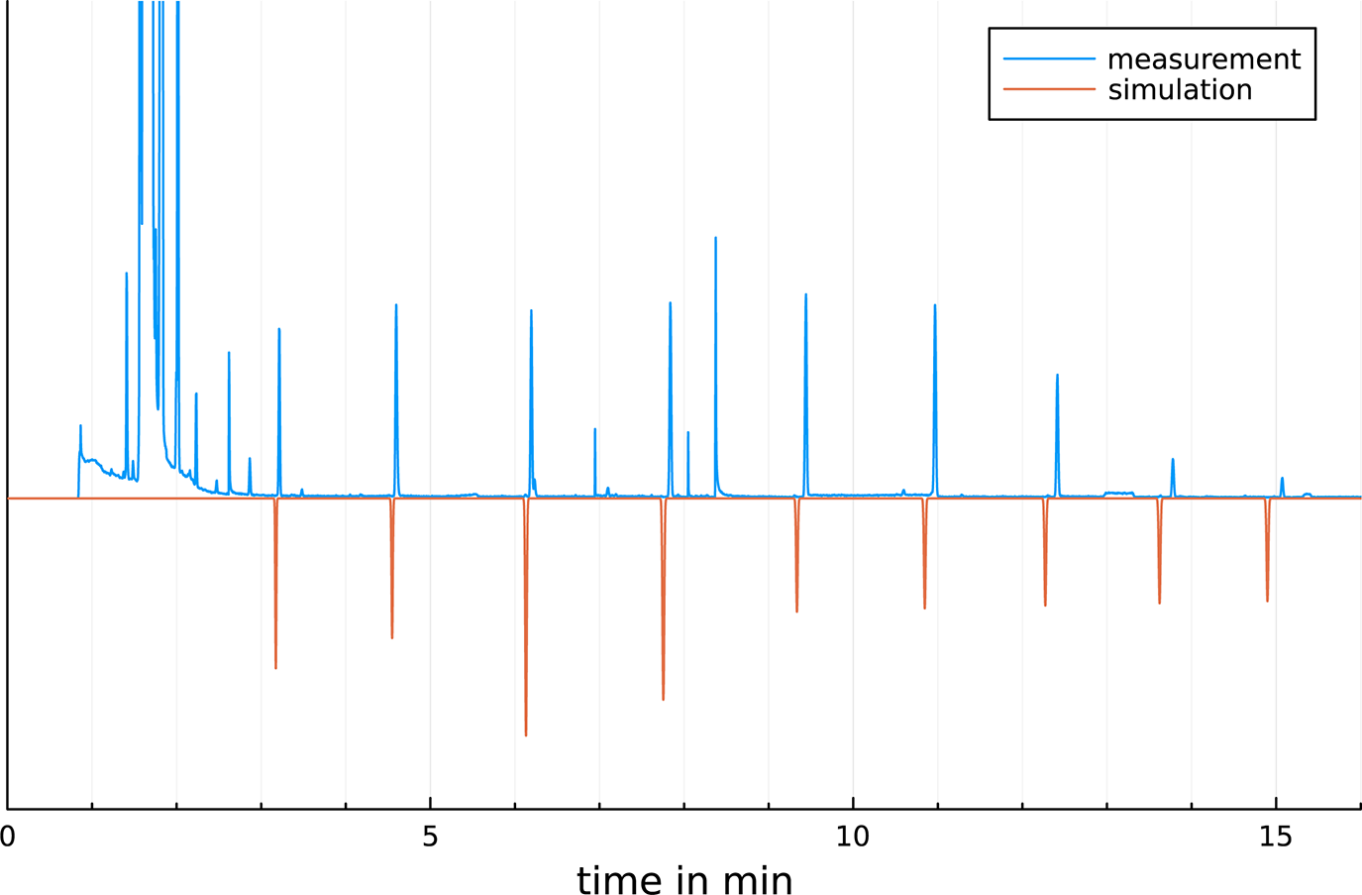
Measured chromatogram of n-alkanes (C8–C20) on a Rxi5SilMS compared to simulation by using ABC retention parameters from Gaida et al.^14^ on Rxi5. GC conditions: *T*_init_ = 40 °C, first ramp: 10 °C/min, *T*_end_ = 300 °C, rmse = 0.2646 min.

The data can be used for prediction and simulation of GC separations. The determined characteristic temperatures of the substances can be directly used to estimate the general elution order of a composition. Most compounds elute in order of their characteristic temperatures. For close *T*_char_ values, the values of θ_char_ and heating rates also have influence on the elution order.^4^ Simulated chromatograms of PAHs and FAMEs compared to measurements on the same GC system are shown in Figures 6 and 7. As demonstrated the simulations well accords to measurements. The average deviation for each compound is less than 1%. The rmse (root-mean-square error) is 0.1425 min for the PAHs and 0.03532 min for the FAMEs. Figure 8 shows a simulation computed by ABC retention parameter from the literature^14^ on a Rxi5 compared to measurements on our own GC system on a Rxi5SilMS. These two stationary phases are similar but do not have exactly same composition; however, the deviations between the retention times for *n*-alkanes are almost less than 2%, which are almost equivalent to a shift by one to three peak widths. In this case, the data are transferable to different GC systems. To check the transferability of the data from one GC system to another, the authors are highly interested in data from the community to compare retention data for similar compounds and phases. As another example for a transferability, the simulation is also suitable for prediction of fast GC measurements such as FF-TG-GC.^2^ Measurements with PAHs^30^ on a FF-TG-GC system show a good match of elution order but a systematic shift in retention times, which result by a lack of knowledge of the exact gradient profile and the different used GC system. A simulation of FF-TG-GC measurements of PAHs compared to measurements is shown in Supporting Information, Figure S5.

## 5 Conclusions

The retention parameter for a huge number of compounds, for example, allergenic fragrances, PAHs, FAMEs, and other volatile substances were determined and collected in a database. The presented calculation procedure is even suitable for method developers on their own GC systems to generate own databases for simple predictions. The presented database now includes data for more than 280 substances on up to 20 different stationary phases. The full database is available at GitHub https://github.com/JanLeppert/RetentionData.^25^ The data are suitable for prediction, simulation, and optimization of GC separations.

To reduce the elaborate isothermal measurements, further investigations will focus on development of easier estimation methods for the retention parameters than via isothermal measurements. The most important *K*-centric parameter *T*_char_ can be well-estimated from the elution temperature. Similar to the estimations of RI or boiling points from LSER data^7^ from the literature, the other *K*-centric parameters can also be estimated. First results are promising. With suitable optimization algorithms, efficient estimates by simulation will be possible from temperature-programed measurements .

## Supporting Information Available

Plot of Lambert W function, ln *k* values of measurements, 3D plots of retention parameters, chromatograms, and results of PCA (PDF)
Databases described in this work before and after validation, and new data measured without any literature data (XLS)
