# Supplementary material for: Retention Database for Prediction, Simulation, and Optimization of GC Separations
Source: ACS Omega. 2023 May 23;8(22):19708–18. doi: 10.1021/acsomega.3c01348 (PMC10249385; doi:10.1021/acsomega.3c01348)
Supplement: Supplementary file 1 — ao3c01348_si_001.pdf [file ao3c01348_si_001.pdf]

Supplemental Material to

Retention Database for Prediction, Simulation and Optimization of GC Separations

Tillman Brehmer[1,*], Benny Duong[1], Manuela Marquart[1], Luise Friedemann[1,2], Peter J. Faust[1,3], Peter Boeker[1,3], Matthias Wüst[1], Jan Leppert[1]

[1] University of Bonn, Institute of Nutritional and Food Sciences, Food Chemistry, Endenicher Allee 11 - 13, 53115 Bonn, Germany

[2] Hochschule Bonn-Rhein-Sieg, Department for Applied Sciences, Von-Liebig-Straße 20, 53359, Rheinbach, Germany

[3] HyperChrom GmbH Germany, Endenicher Allee 11 -13, 53115, Bonn, Germany

*Corresponding author: Tillman Brehmer, brehmer@uni-bonn.de

Table of Content

Introduction: ... 3

Excel File Databases.xls ... 3

Plot of Lambert W function ... 3

$\ln k$ Values of Measurements ... 3

3D Plots of Retention Parameters ... 4

ABC-Parameters ... 4

K-centric Parameters ... 4

Chromatograms ... 5

Simulation and Temperature programmed GC Measurement of unsaturated FAMEs ... 5

Simulation and Fast GC Measurement of PAH via FF TG GC ... 6

Results of Principal Compound Analyses ... 7

Results of PCA for ABC parameters ... 7

Results of PCA for K-centric parameters ... 7

Static Pluto Notebook "Notebook PCA" ... 7

Introduction:

The supporting information includes additional 3D plots of the retention parameters, statistic data of the PCA analyses, chromatograms of unsaturated FAMEs fast GC measurement of PAH.

Excel File Databases.xls

The database described in the article is separately given as Excel file “Databases.xls”. There you can find three tables: First with all Data before the validation described in the article, second after validation, third with only the data, that are new measured for this publication without any literature data.

The Database is also available via: https://github.com/JanLeppert/RetentionData/blob/main/Databases/database_all.csv. There you also can find the csv files of the database for machine reading.

Have Fun!

Plot of Lambert W function

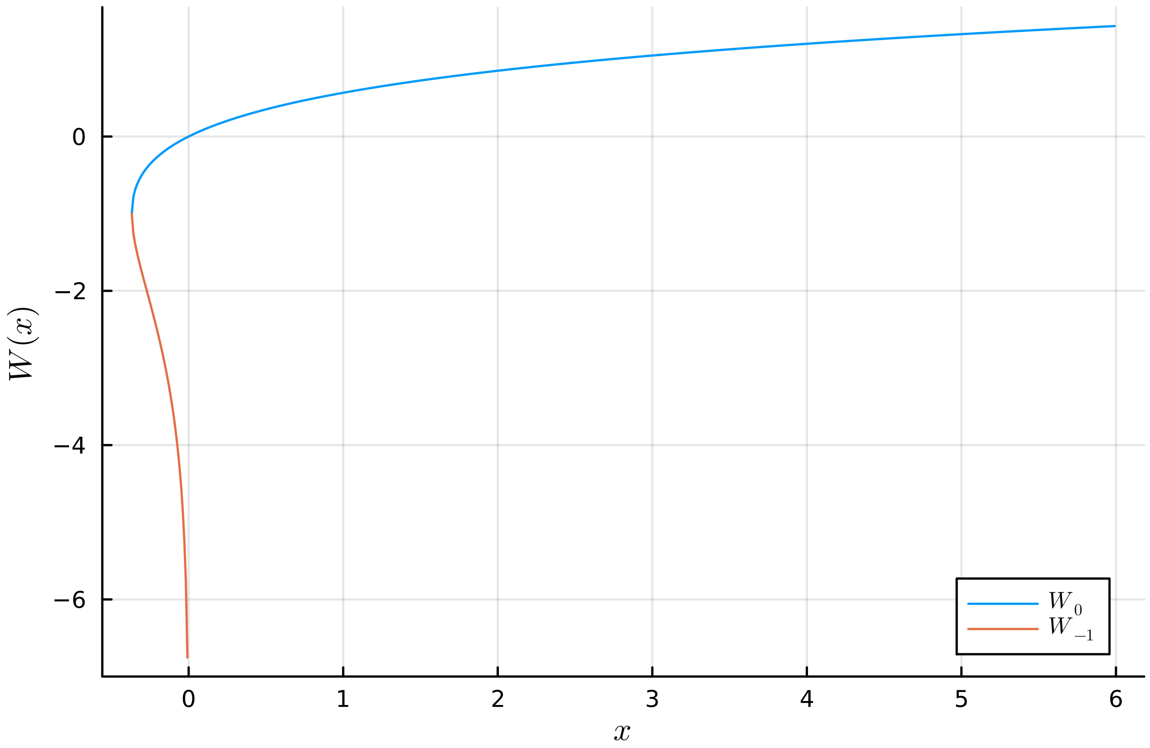

Figure S1 Plot of the Lambert W function with its to branches W_0 and W_{-1}.

$\ln k$ Values of Measurements

The $\ln k$ values of all measured compounds are available via GitHub at: https://github.com/JanLeppert/RetentionData/tree/main/Databases/Measurements

3D Plots of Retention Parameters

ABC-Parameters

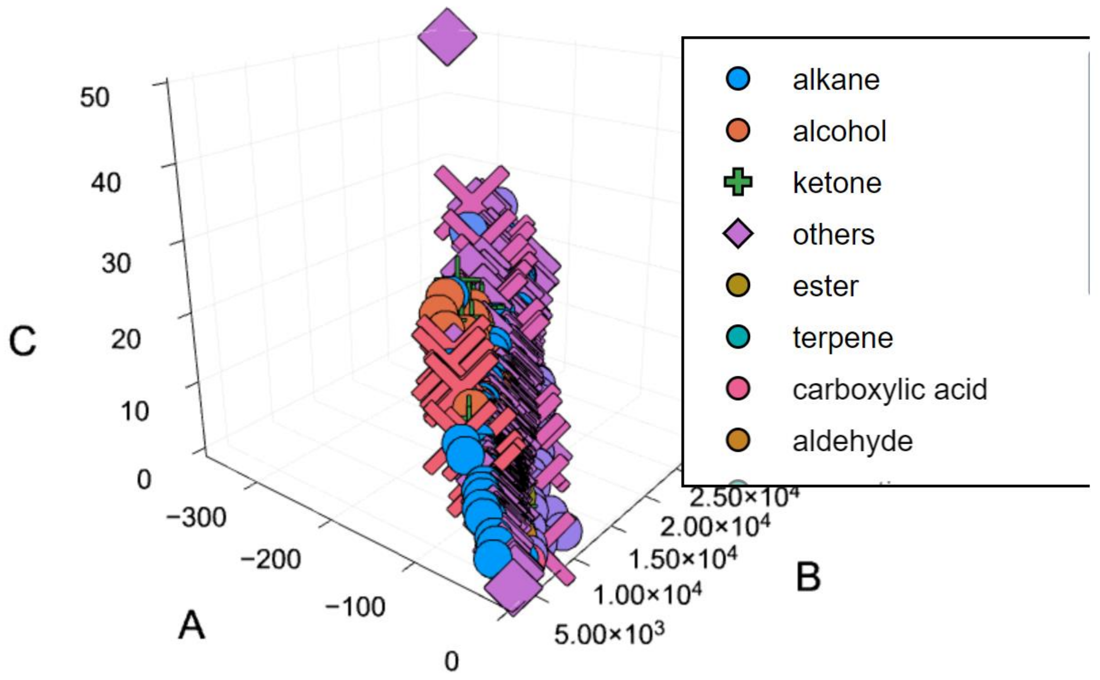

Figure S2: 3D plot of the ABC parameters compound category. For interactive view go to https://raw.githack.com/JanLeppert/RetentionData/main/Supplemental%20Materials/FigureS2.html

K-centric Parameters

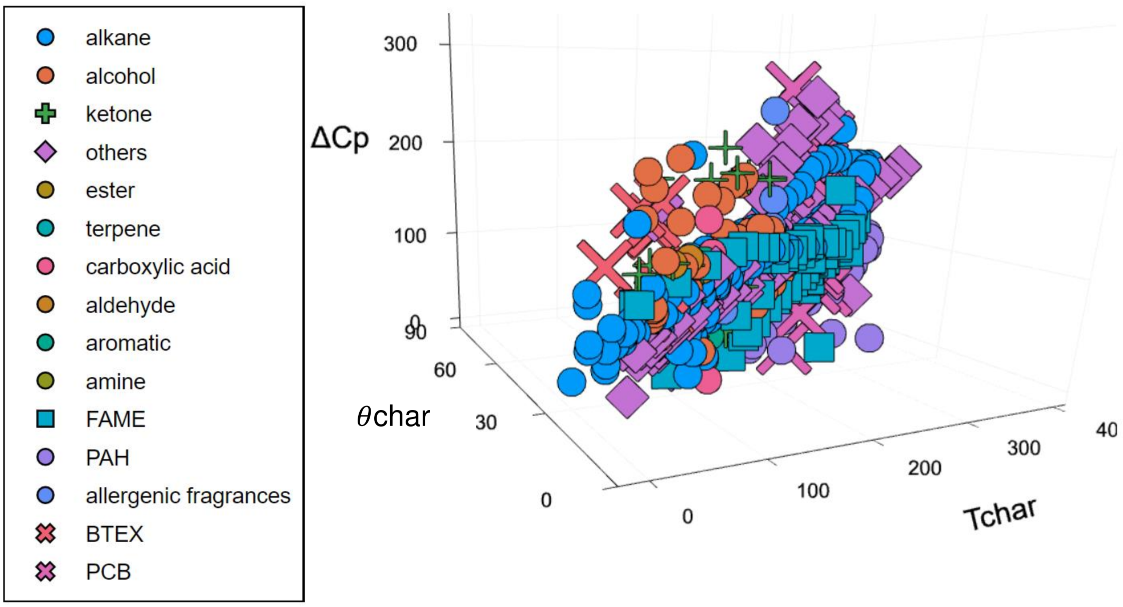

Figure S3: 3D plot of the K-centric parameters sorted by compound category. For interactive view go to https://raw.githack.com/JanLeppert/RetentionData/main/Supplemental%20Materials/FigureS3.html

Chromatograms

Simulation and Temperature programmed GC Measurement of unsaturated FAMEs

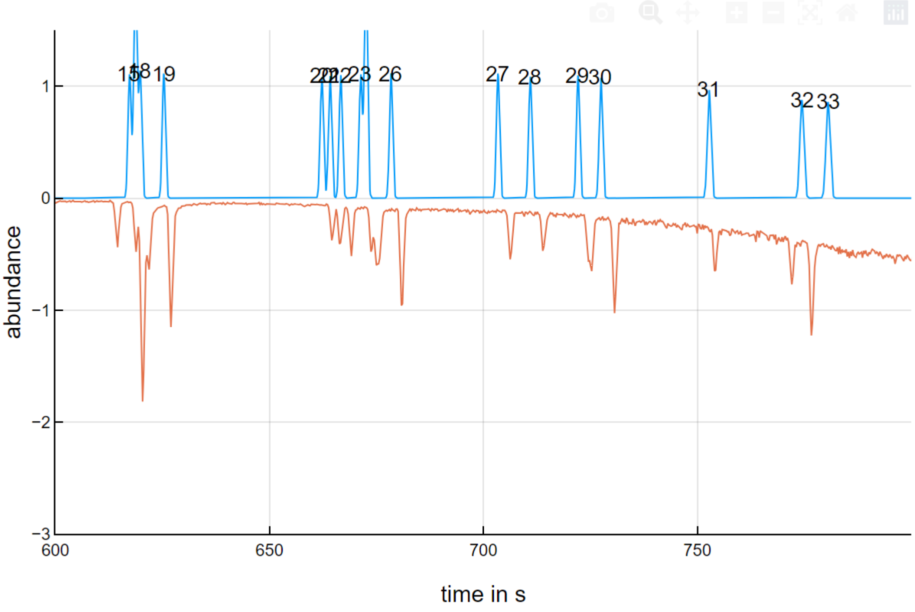

Figure S4: Chromatogram of the measurement and simulation of FAMEs with focus on the simulation of unsaturated FAMES such as C18:1, C18:2, C18:3 and C20 derivates. For interactive view go to *https://raw.githack.com/JanLeppert/RetentionData/main/Supplemental%20Materials/FigureS4.html*

Simulation and Fast GC Measurement of PAH via FF TG GC

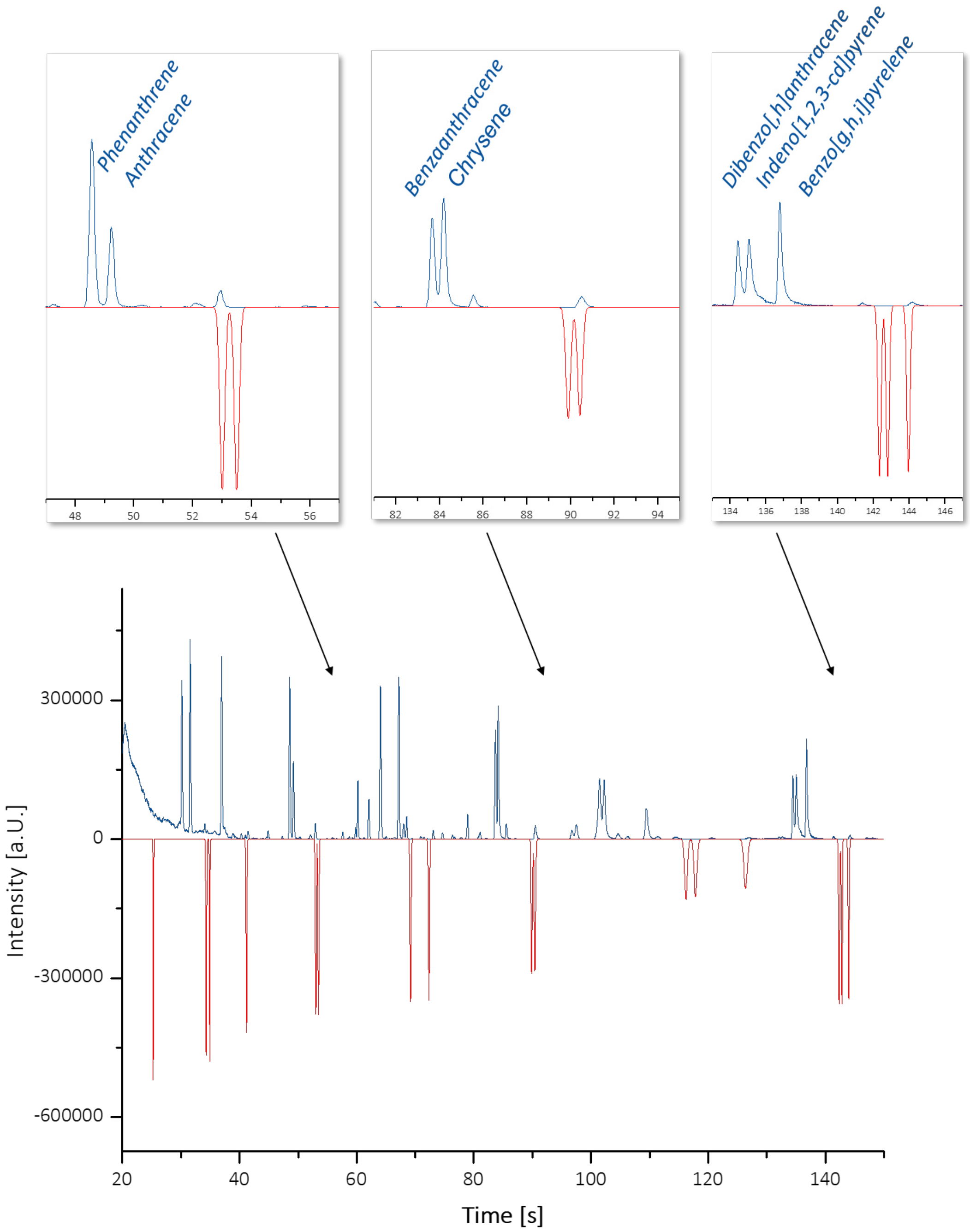

Figure S5: Comparison between simulated (red) and measured (blue) chromatogram of Flow Field Thermal Gradient (FF TG GC) separation of 16 EPA PAH on ZB-PAH-CT column (4 m, β=0.001).

Results of Principal Compound Analyses

Important statistic data are shown below. The whole static Pluto notebook “Notebook_PCA.html”, including the Database, some 3D plots of the data and processing steps of the PCA analysis can be found on GitHub via the link: https://github.com/JanLeppert/RetentionData/blob/main/Supplemental%20Materials/Notebook_PCA.jl.html. Use the Table of Content menu on the right hand side to navigate through the document.

Results of PCA for ABC parameters

PCA(indim = 3, outdim = 1, principalratio = 0.9999847366292303)

Pattern matrix (unstandardized loadings):

	PC1
1	-36.8846
2	4402.83.
3	4.79112

Importance of components:

	PC1
SS Loadings (Eigenvalues)	1.93863 e7
Variance explained	0.999985
Cumulative variance	0.999985
Proportion explained	1.0
Cumulative proportion	1.0

Results of PCA for K-centric parameters

PCA(indim = 3, outdim = 2, principalratio = 0.9965619007969799)

Pattern matrix (unstandardized loadings):

	PC1	PC2
1	78.4105	-12.7126
2	5.44017	-0.253663
3	28.0469	35.5897

Importance of components:

	PC1	PC2
SS Loadings (Eigenvalues)	6964.43	1428.31
Variance explained	0.826964	0.169598
Cumulative variance	0.826964	0.170183
Proportion explained	0.826964	0.170183
Cumulative proportion	0.826964	1.0

Static Pluto Notebook “Notebook PCA”

The following pages show the static notebook “Notebook_PCA.jl.html”. For interactive view in your browser open the link above or run the notebook with Julia.

Supplemental Materials - Investigations and PCA Analysises

Table of Contents

Supplemental Materials - Investigations and PCA Analysises

Load `AllParam` data

Plot parameters
- Plot parameters Tchar, θchar, ΔCp
- Plot parameters A, B, C
- Plot parameters ΔHchar, ΔSchar, ΔCp
- Plot parameters ΔHref, ΔSref, ΔCp

Flagged substances
- Plot parameters Tchar, θchar, ΔCp
- Plot parameters A, B, C
- Plot parameters ΔHchar, ΔSchar, ΔCp
- Plot parameters ΔHref, ΔSref, ΔCp

Investigate Compound Influence
- Filter functions
- ABC Model
- K-centric Retention Parameter
- Dependence between θchar and Tchar

PCA Analysis
- PCA ABC Model
- PCA K-centric Model

END

Load `AllParam` data

- Load all files with `AllParam` from the folder `db_path=` C:\Users\Brehmer\Documents\GitHub\RetentionData\Databases (and its subfolders).
- use ChemicalIdentifiers.jl
- combine all data into one DataFrame/csv-file

	Name	Phase	Source	DataSet
1	"C7"	"DB5ms"	"Boswell2012"	"Boswell2012_AllParam_TabelS1_DB5
2	"Propyl acetate"	"DB5ms"	"Boswell2012"	"Boswell2012_AllParam_TabelS1_DB5
3	"Toluene "	"DB5ms"	"Boswell2012"	"Boswell2012_AllParam_TabelS1_DB5
4	"Pyridine"	"DB5ms"	"Boswell2012"	"Boswell2012_AllParam_TabelS1_DB5
5	"Butyl acetate"	"DB5ms"	"Boswell2012"	"Boswell2012_AllParam_TabelS1_DB5
6	"C8"	"DB5ms"	"Boswell2012"	"Boswell2012_AllParam_TabelS1_DB5
7	"Pentyl acetate "	"DB5ms"	"Boswell2012"	"Boswell2012_AllParam_TabelS1_DB5
8	"2-Hexanone"	"DB5ms"	"Boswell2012"	"Boswell2012_AllParam_TabelS1_DB5
9	"Ethylbenzene"	"DB5ms"	"Boswell2012"	"Boswell2012_AllParam_TabelS1_DB5
10	"2-Heptanone"	"DB5ms"	"Boswell2012"	"Boswell2012_AllParam_TabelS1_DB5
more				
1031	"2- phenoxyphenol"	"ZB-XLB-HT"	"Ulrich2012"	"Ulrich2012_AllParam_TableS4_ZB-X

```
begin
    alldata = RetentionData.dataframe_of_all(data)
    # add flags to alldata
    alldata[!, "flag"] = RetentionData.flag(alldata)
    CI = RetentionData.substance_identification(alldata)
    # add CAS to alldata
    alldata[!, "CAS"] = CI.CAS
    fl, nfl = RetentionData.flagged_data(alldata)
    alldata
end
```

Plot parameters

3D-Plots of the parameters of one parameter set. Only unflagged substances are used.

```
md"""
# Plot parameters

3D-Plots of the parameters of one parameter set. Only unflagged substances are used.
"""
```

Plot parameters Tchar, θchar, ΔCp

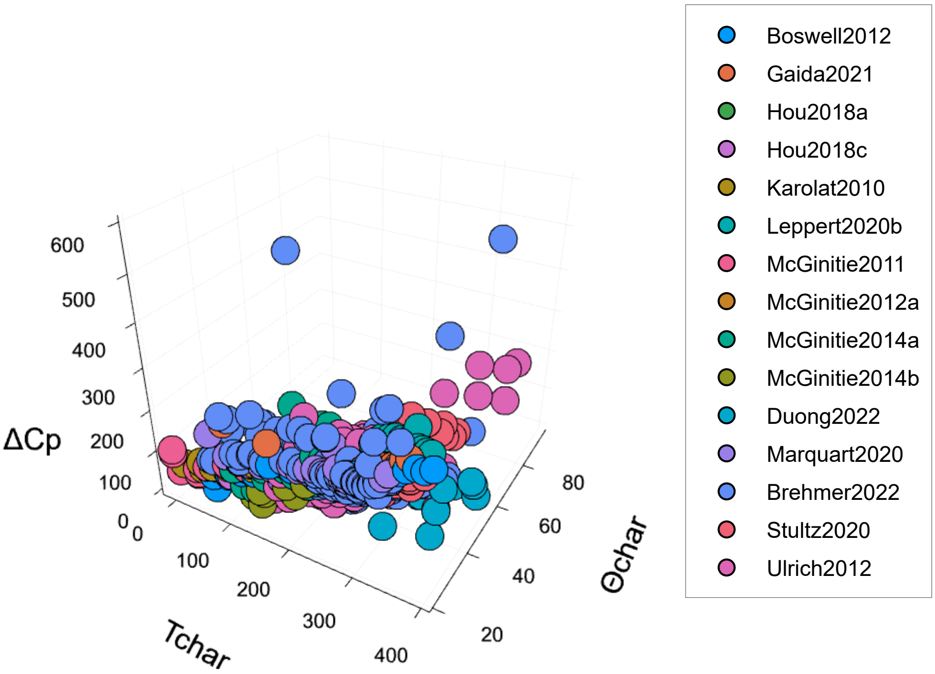


```
For saving to png with the `Plotly` backend `PlotlyBase` and `PlotlyKaleido` ne
ed to be installed.
err:
    ArgumentError("Package PlotlyBase not found in cur" … 53 bytes … "to insta
```

Plot parameters A, B, C

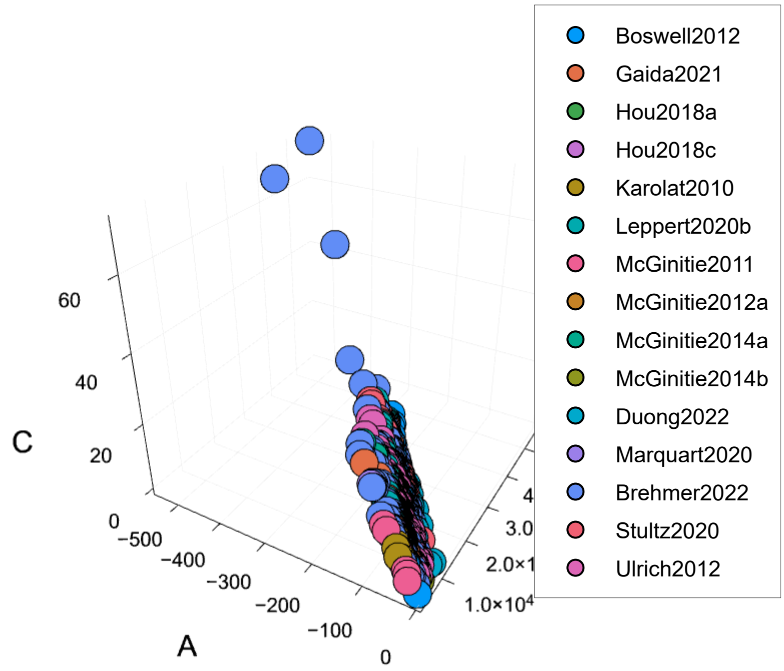

Plot parameters ΔHchar, ΔSchar, ΔCp

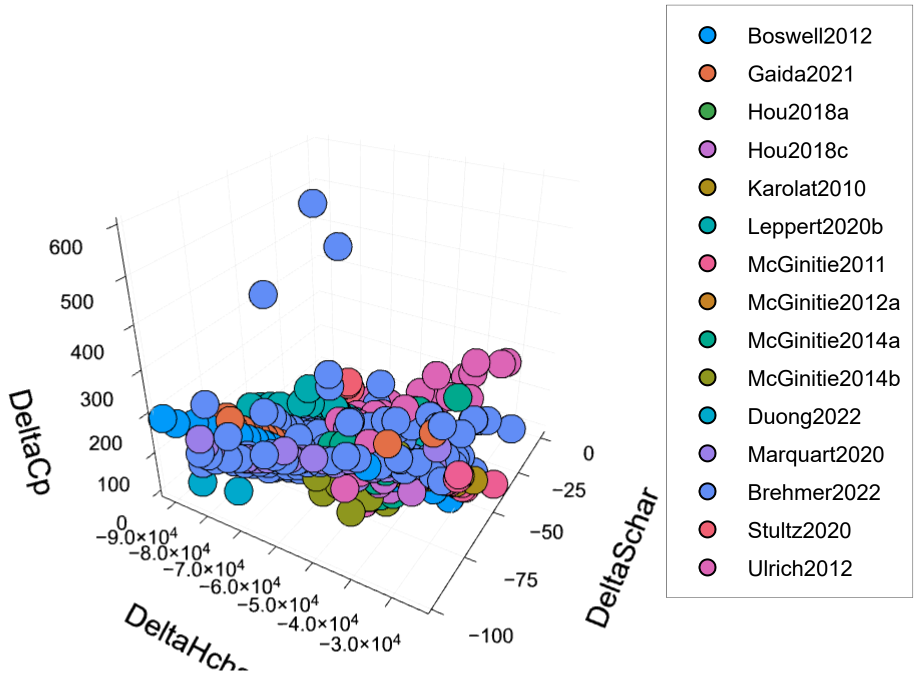

Plot parameters ΔHref, ΔSref, ΔCp

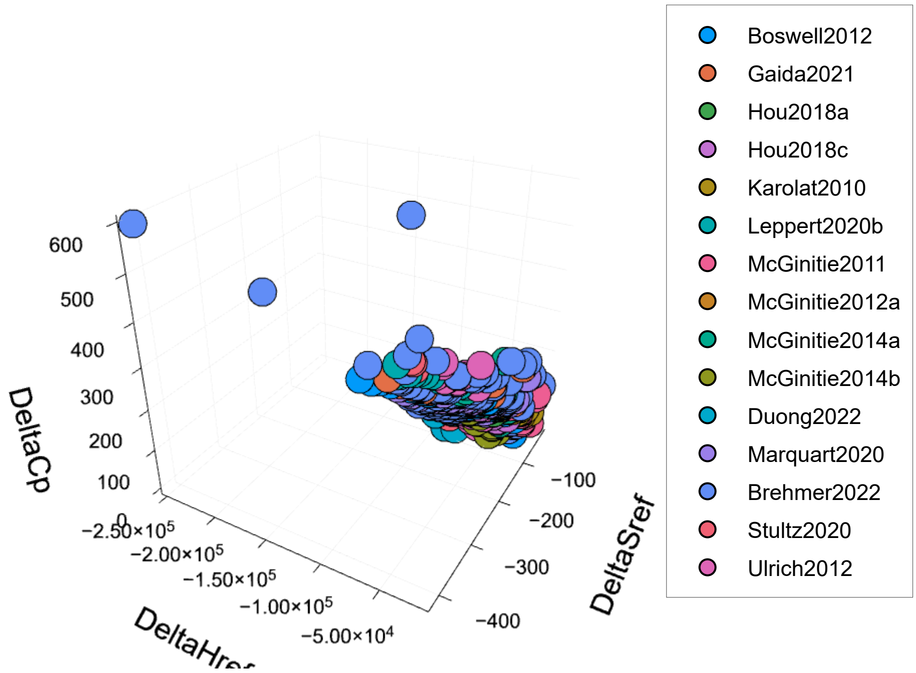

Flagged substances

Flagged substances are added to the 3D-plots of the parameter sets as red crosses.

Plot parameters Tchar, θchar, ΔCp

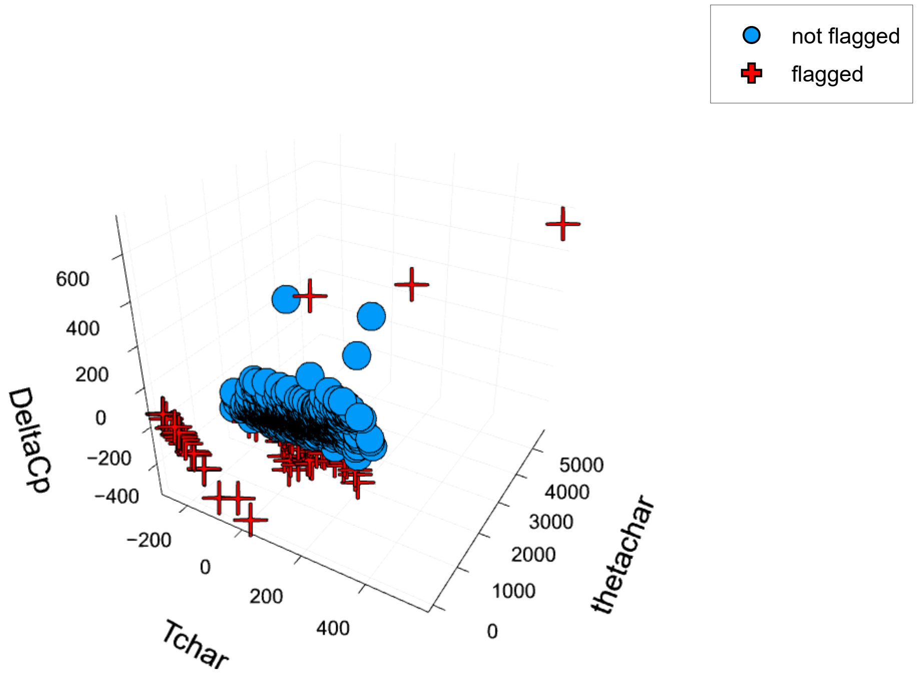

Plot parameters A, B, C

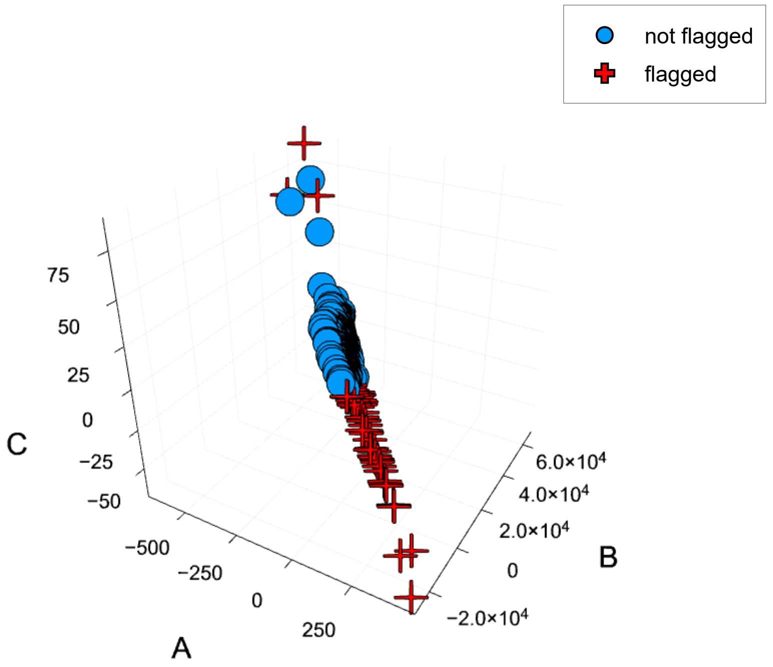

Plot parameters ΔHchar, ΔSchar, ΔCp

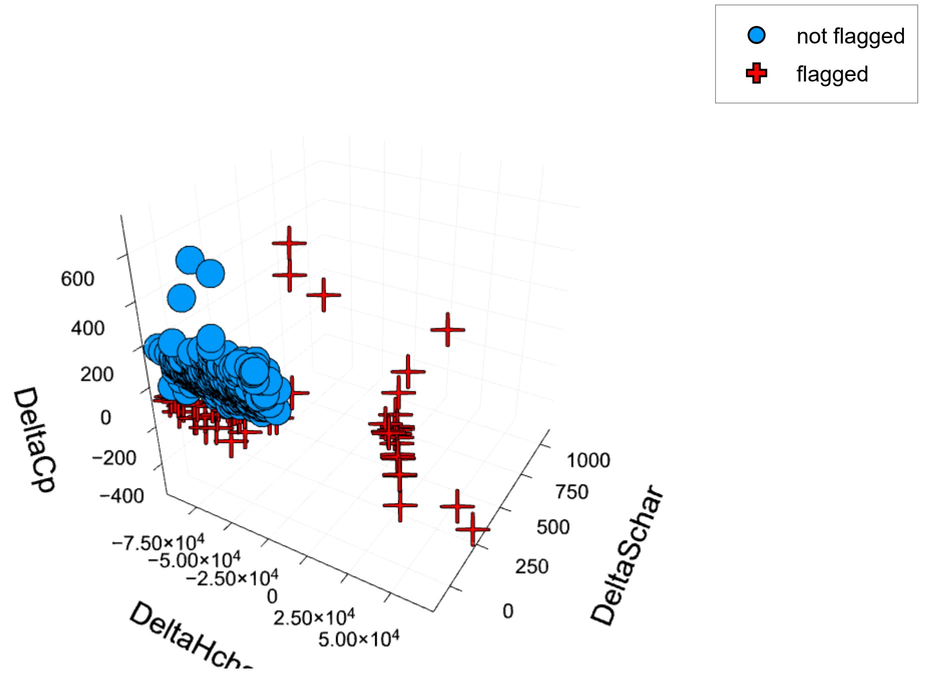

Plot parameters ΔHref, ΔSref, ΔCp

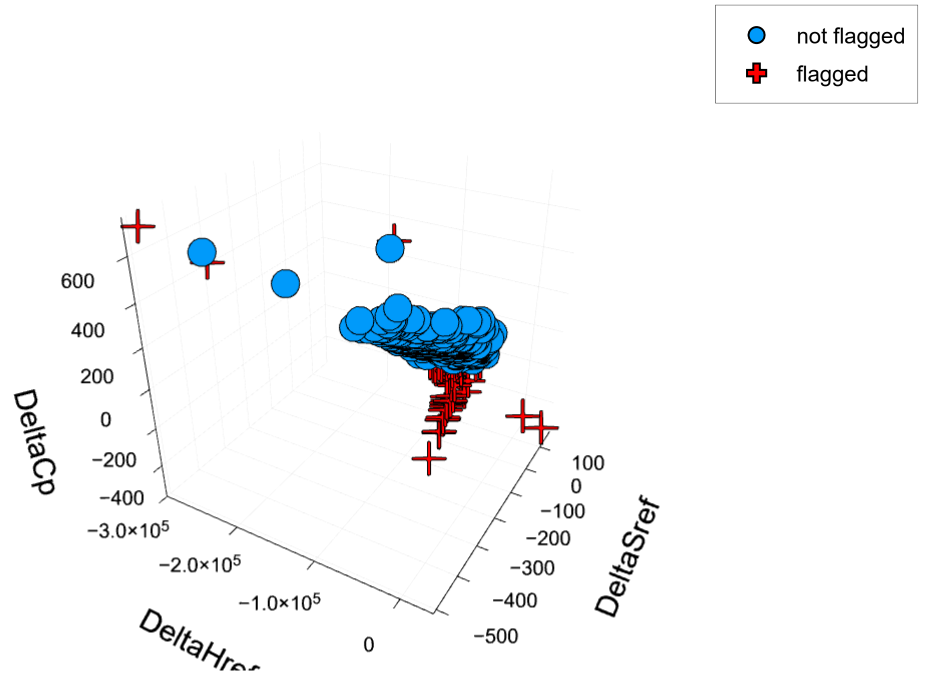

Investigate Compound Influence

Can we found systematical influences of the compound category to the parameters?

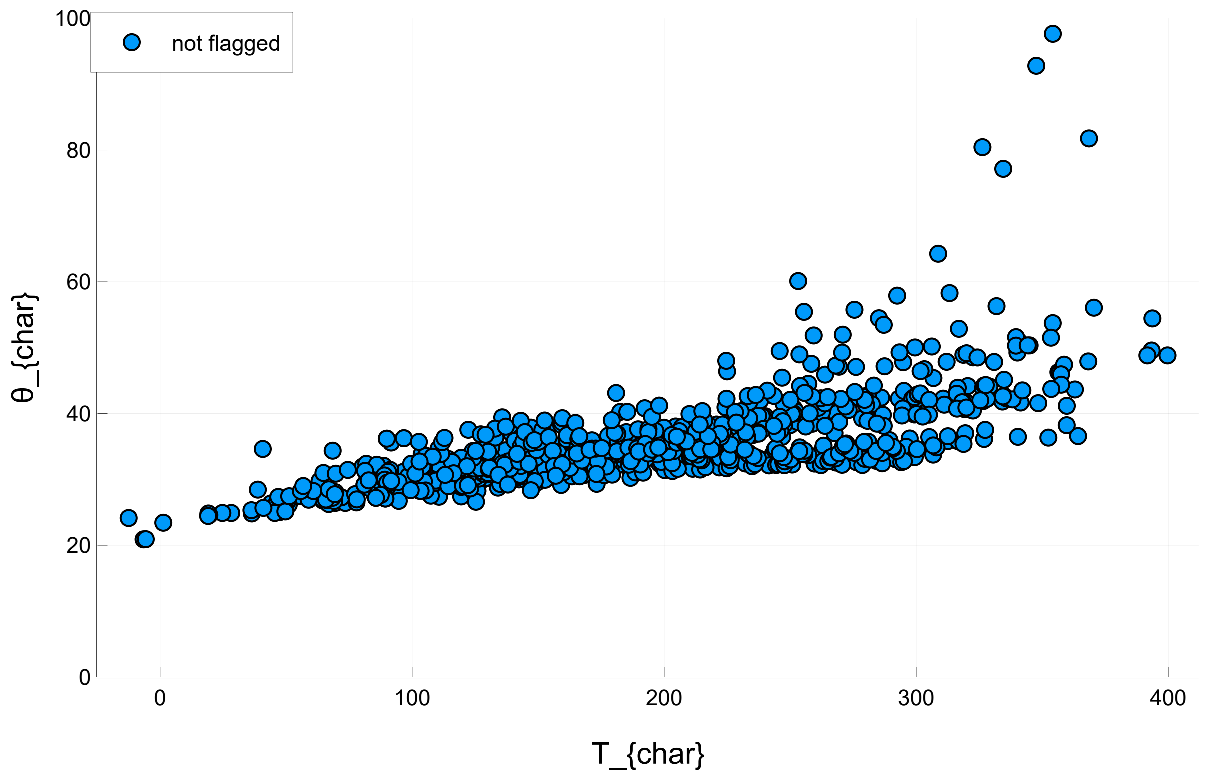

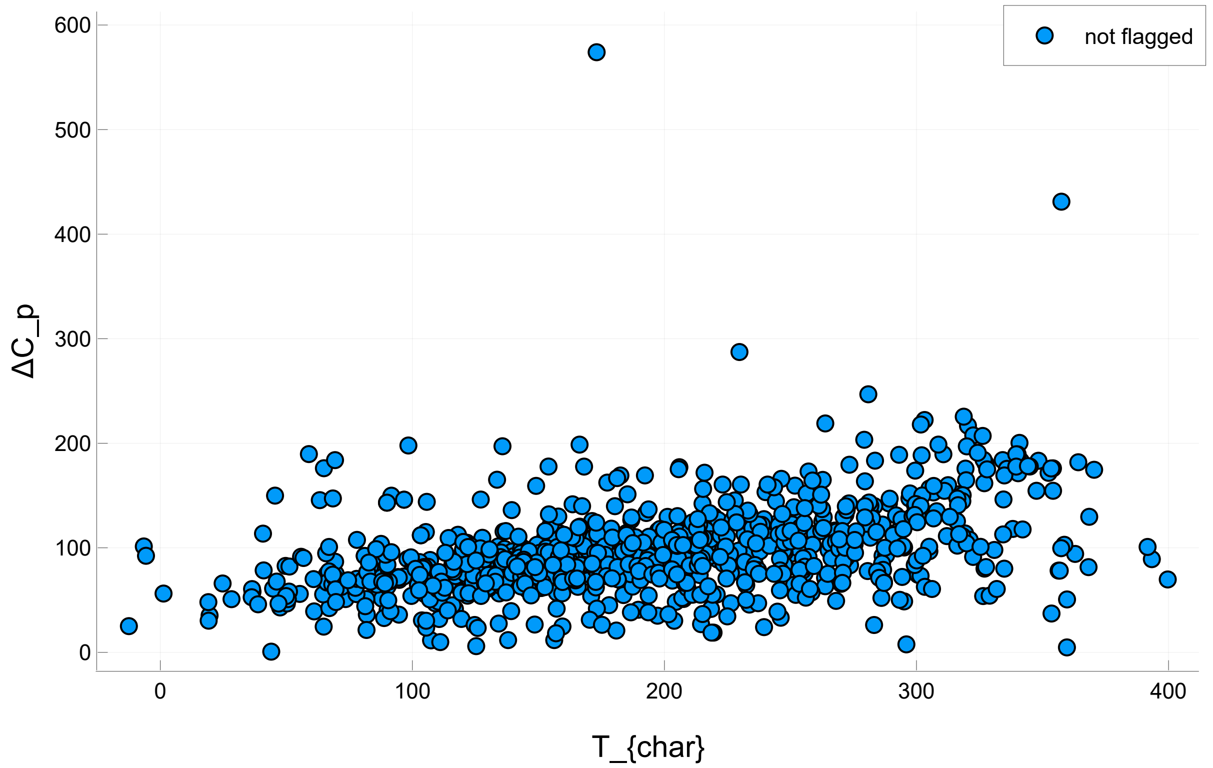

Filter functions

```
SubstanceFilter =
```

	Name	Phase	Source	DataSet
1	"C7"	"DB5ms"	"Boswell2012"	"Boswell2012_AllParam_TabelS1_D
2	"Propyl acetate"	"DB5ms"	"Boswell2012"	"Boswell2012_AllParam_TabelS1_D
3	"Toluene "	"DB5ms"	"Boswell2012"	"Boswell2012_AllParam_TabelS1_D
4	"Pyridine"	"DB5ms"	"Boswell2012"	"Boswell2012_AllParam_TabelS1_D
5	"Butyl acetate"	"DB5ms"	"Boswell2012"	"Boswell2012_AllParam_TabelS1_D
6	"C8"	"DB5ms"	"Boswell2012"	"Boswell2012_AllParam_TabelS1_D
7	"Pentyl acetate "	"DB5ms"	"Boswell2012"	"Boswell2012_AllParam_TabelS1_D
8	"2-Hexanone"	"DB5ms"	"Boswell2012"	"Boswell2012_AllParam_TabelS1_D
9	"Ethylbenzene"	"DB5ms"	"Boswell2012"	"Boswell2012_AllParam_TabelS1_D
10	"2-Heptanone"	"DB5ms"	"Boswell2012"	"Boswell2012_AllParam_TabelS1_D
	more			
394	"2- phenoxyphenol"	"ZB-XLB-HT"	"Ulrich2012"	"Ulrich2012_AllParam_TableS4_ZB

ABC Model

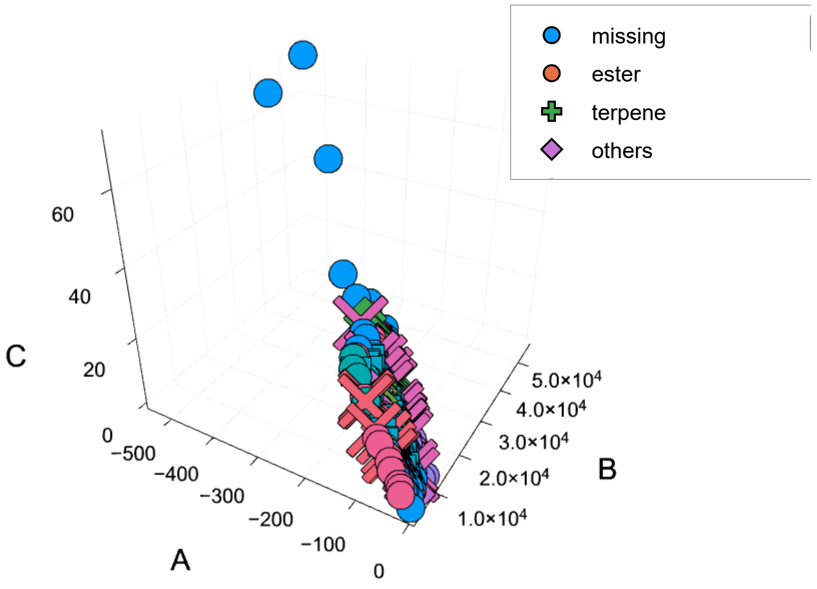

K-centric Retention Parameter

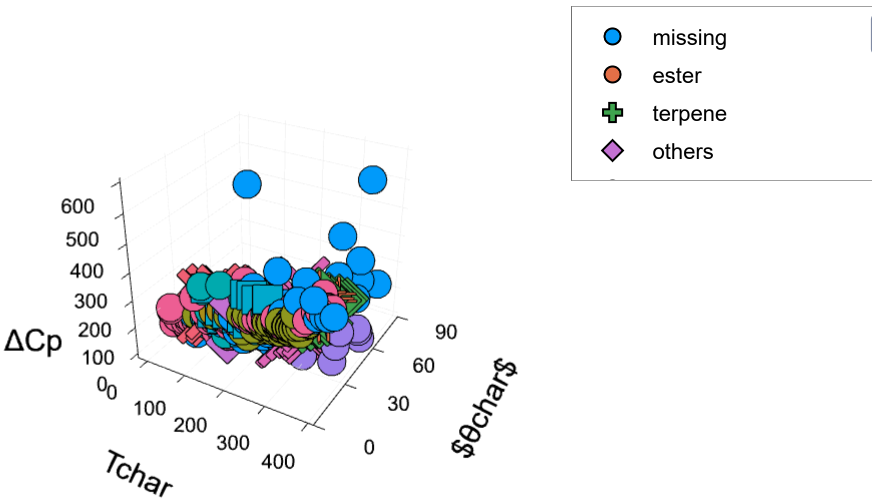


```
Indices Base.OneTo(1) of attribute `seriescolor` does not match data indices 1:394.

Indices Base.OneTo(1) of attribute `linecolor` does not match data indices 1:394.

Indices Base.OneTo(1) of attribute `fillcolor` does not match data indices 1:394.

Indices Base.OneTo(1) of attribute `markercolor` does not match data indices 1:394.

Indices Base.OneTo(1) of attribute `seriescolor` does not match data indices 1:5.

Indices Base.OneTo(1) of attribute `linecolor` does not match data indices 1:5.

Indices Base.OneTo(1) of attribute `fillcolor` does not match data indices 1:5.

Indices Base.OneTo(1) of attribute `markercolor` does not match data indices 1:5.

Indices Base.OneTo(1) of attribute `seriescolor` does not match data indices 1:5.

Indices Base.OneTo(1) of attribute `linecolor` does not match data indices 1:5.

Indices Base.OneTo(1) of attribute `fillcolor` does not match data indices 1:5.
```

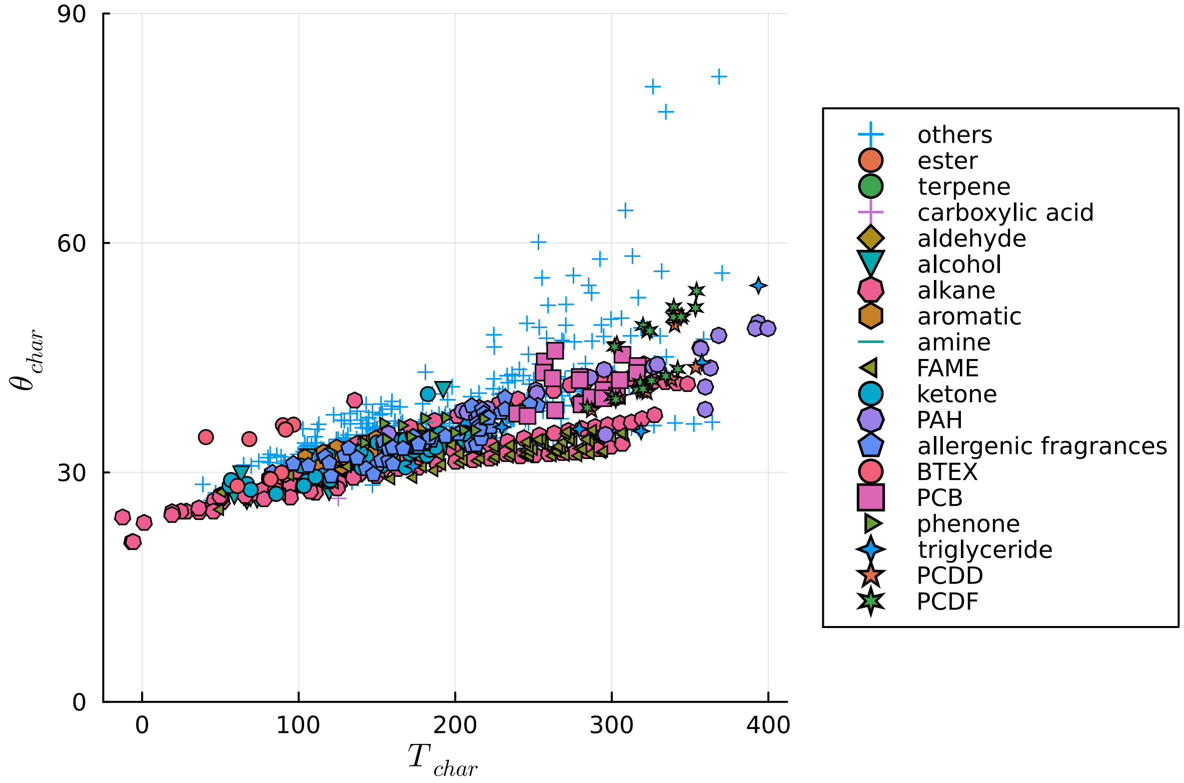


```
Skipped marker arg pixel.

Indices Base.OneTo(1) of attribute `seriescolor` does not match data indices
1:394.

Indices Base.OneTo(1) of attribute `linecolor` does not match data indices 1:
394.

Indices Base.OneTo(1) of attribute `fillcolor` does not match data indices 1:
394.

Indices Base.OneTo(1) of attribute `markercolor` does not match data indices
1:394.

Indices Base.OneTo(1) of attribute `seriescolor` does not match data indices
1:394.

Indices Base.OneTo(1) of attribute `linecolor` does not match data indices 1:
394.

Indices Base.OneTo(1) of attribute `fillcolor` does not match data indices 1:
394.

Indices Base.OneTo(1) of attribute `markercolor` does not match data indices
1:394.

Indices Base.OneTo(1) of attribute `seriescolor` does not match data indices
1:5.

Indices Base.OneTo(1) of attribute `linecolor` does not match data indices 1:
5.

Indices Base.OneTo(1) of attribute `fillcolor` does not match data indices 1:
```

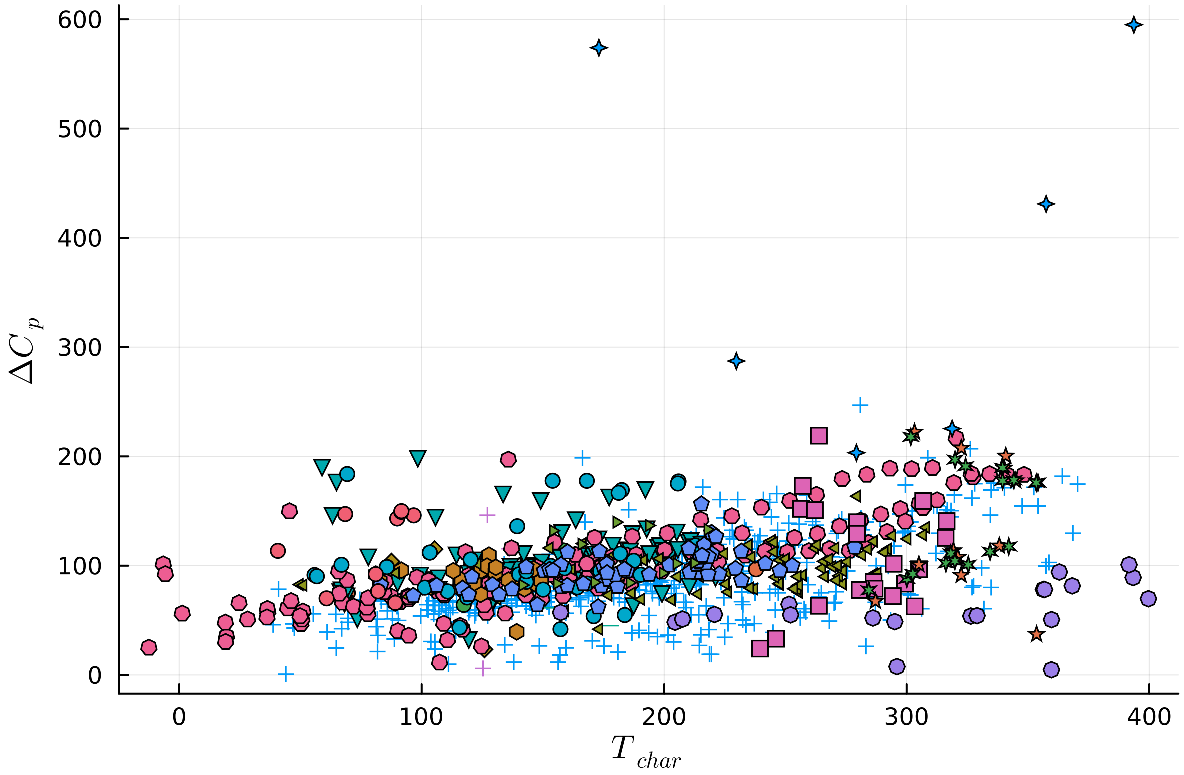


```
Skipped marker arg pixel.
```

Dependence between θchar and Tchar

Fit Blumberg2022/2010 Model

$$\theta_{char} = (T_{char})^{0.7} \cdot (10^3 \cdot \varphi)^{0.09}$$

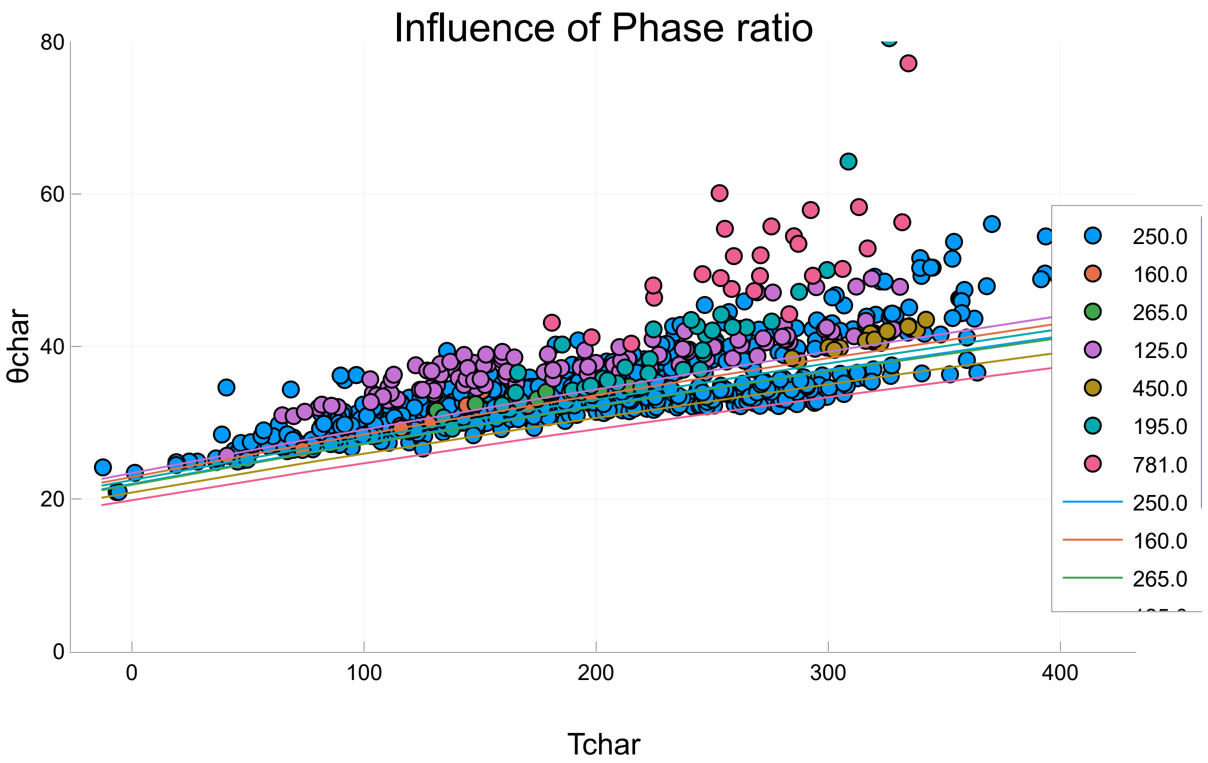

Spread of θ_{char} for the same phase ratio is higher (points) than the difference between the phase ration (lines).

PCA Analysis

```
using MultivariateStats    , RDatasets
```

PCA ABC Model

	Name	Phase	Source	
1	"C7"	"DB5ms"	"Boswell2012"	"Boswell
2	"Propyl acetate"	"DB5ms"	"Boswell2012"	"Boswell
3	"Toluene "	"DB5ms"	"Boswell2012"	"Boswell
4	"Pyridine"	"DB5ms"	"Boswell2012"	"Boswell
5	"Butyl acetate"	"DB5ms"	"Boswell2012"	"Boswell
6	"C8"	"DB5ms"	"Boswell2012"	"Boswell
7	"Pentyl acetate "	"DB5ms"	"Boswell2012"	"Boswell
8	"2-Hexanone"	"DB5ms"	"Boswell2012"	"Boswell
9	"Ethylbenzene"	"DB5ms"	"Boswell2012"	"Boswell
10	"2-Heptanone"	"DB5ms"	"Boswell2012"	"Boswell
11	"C9"	"DB5ms"	"Boswell2012"	"Boswell
12	"Hexyl acetate"	"DB5ms"	"Boswell2012"	"Boswell
13	"2-Octanone"	"DB5ms"	"Boswell2012"	"Boswell
14	"C10"	"DB5ms"	"Boswell2012"	"Boswell
15	"N,N-dimethylisobutyramide "	"DB5ms"	"Boswell2012"	"Boswell

```
nfl
```

index_ABC = [5, 7, 9]

```
index_ABC = [5, 7, 9]
```

index_Kcentric = [11, 13, 15]

```
index_Kcentric = [11, 13, 15]
```

```
ABC_Training =
3×483 adjoint(::Matrix{Float64}) with eltype Float64:
   -7.0955    -38.39     -49.995    -59.64    …   -73.538     -62.588    -100.14
 3843.9      5512.4     6575.7     7531.4        11672.0     10307.0     12493.0
    0.08624     4.7175     6.2243     7.5351         9.0387      7.6019     12.929
```

```
ABC_Training=Matrix(nfl[1:2:end-1,index_ABC])'
```

```
ABC_Testing =
3×483 adjoint(::Matrix{Float64}) with eltype Float64:
  -42.291    -52.142    -41.857    -52.602    …    -65.317    -115.38     -97.084
 5763.8     6137.6     6016.3     6510.3        10348.0     14022.0    12452.0
    5.1709     6.7269     5.0936     6.6723          7.9913     14.949     12.54
```

```
ABC_Testing=Matrix(nfl[2:2:end,index_ABC])'
```

```
PCA_ABC = PCA(indim = 3, outdim = 1, principalratio = 0.9999847366292303)

          Pattern matrix (unstandardized loadings):
          ─────────────
                   PC1
          ─────────────
          1   -36.8846
          2  4402.83
          3     4.79112
          ─────────────

          Importance of components:
          ────────────────────────────────────
                                          PC1
          ────────────────────────────────────
          SS Loadings (Eigenvalues)  1.93863e7
          Variance explained         0.999985
          Cumulative variance        0.999985
          Proportion explained       1.0
          Cumulative proportion      1.0
          ────────────────────────────────────
```

```
PCA_ABC=fit(PCA, ABC_Training; maxoutdim=2)
```

```
ABC_Y =
1×483 Matrix{Float64}:
 6024.28  5650.41  5771.79  5277.71  4649.71  …  3667.31  1440.04  -2234.25  -664.152
```

```
ABC_Y=predict(PCA_ABC,  ABC_Testing)
```

```
3×483 Matrix{Float64}:
  -41.578    -44.71     -43.6931   …    -79.981    -110.761     -97.6082
 5763.81    6137.66    6016.28        10347.9     14022.0     12452.0
    5.22054    5.62736    5.49528        10.2089     14.2071     12.4986
```

```
reconstruct(PCA_ABC, ABC_Y)
```

PCA K-centric Model

```
kcentric_Training =
3×483 adjoint(::Matrix{Float64}) with eltype Float64:
 43.997    61.023  69.587  89.881  81.927  …  228.64   243.51   217.36   214.07
 26.354    28.373  26.442  27.48   29.592      38.615   38.12    36.575   38.329
  0.71704  39.223  51.751  62.651  36.157     124.3     75.152   63.205  107.5
```

```
kcentric_Training=Matrix(nfl[1:2:end-1,index_Kcentric])'
```

```
kcentric_Testing =
3×483 adjoint(::Matrix{Float64}) with eltype Float64:
 47.513  55.332  67.026  64.593  85.857  …  218.69   208.87   228.64   236.33
 25.045  27.47   27.015  26.797  27.986      34.547   35.767   38.615   42.813
 42.993  55.93   42.351  55.477  58.663      18.91    66.444  124.3    104.26
```

```
kcentric_Testing=Matrix(nfl[2:2:end,index_Kcentric])'
```

```
PCA_kcentric = PCA(indim = 3, outdim = 2, principalratio = 0.9965619007969799)

Pattern matrix (unstandardized loadings):
──────────────────────
        PC1        PC2
──────────────────────
1  78.4105   -12.7126
2   5.44017   -0.253663
3  28.0469    35.5897
──────────────────────

Importance of components:
───────────────────────────────────────────────────
                                   PC1         PC2
───────────────────────────────────────────────────
SS Loadings (Eigenvalues)  6964.43      1428.31
Variance explained            0.826964     0.169598
Cumulative variance           0.826964     0.996562
Proportion explained          0.829817     0.170183
Cumulative proportion         0.829817     1.0
───────────────────────────────────────────────────
```

```
PCA_kcentric=fit(PCA, kcentric_Training; maxoutdim=2)
```

```
kcentric_Y =
2×483 Matrix{Float64}:
 151.604    139.751    133.357   131.246    110.118  …   -8.58375  -46.789    -47.553
  -4.21776    5.31866  -11.3993    1.78141   -2.379     -36.4824    11.3315  -10.1551
```

```
kcentric_Y= predict(PCA_kcentric, kcentric_Testing)
```

```
3×483 Matrix{Float64}:
 47.4897  55.4181  67.0494  64.5993  …  218.601   208.851   228.664   236.609
 25.4109  26.1196  26.6486  26.6978      35.9473   36.0698   38.2394   38.4335
 42.9873  55.9511  42.3567  55.4786      18.8881   66.4393  124.306   104.329
```

```
reconstruct(PCA_kcentric, kcentric_Y)
```

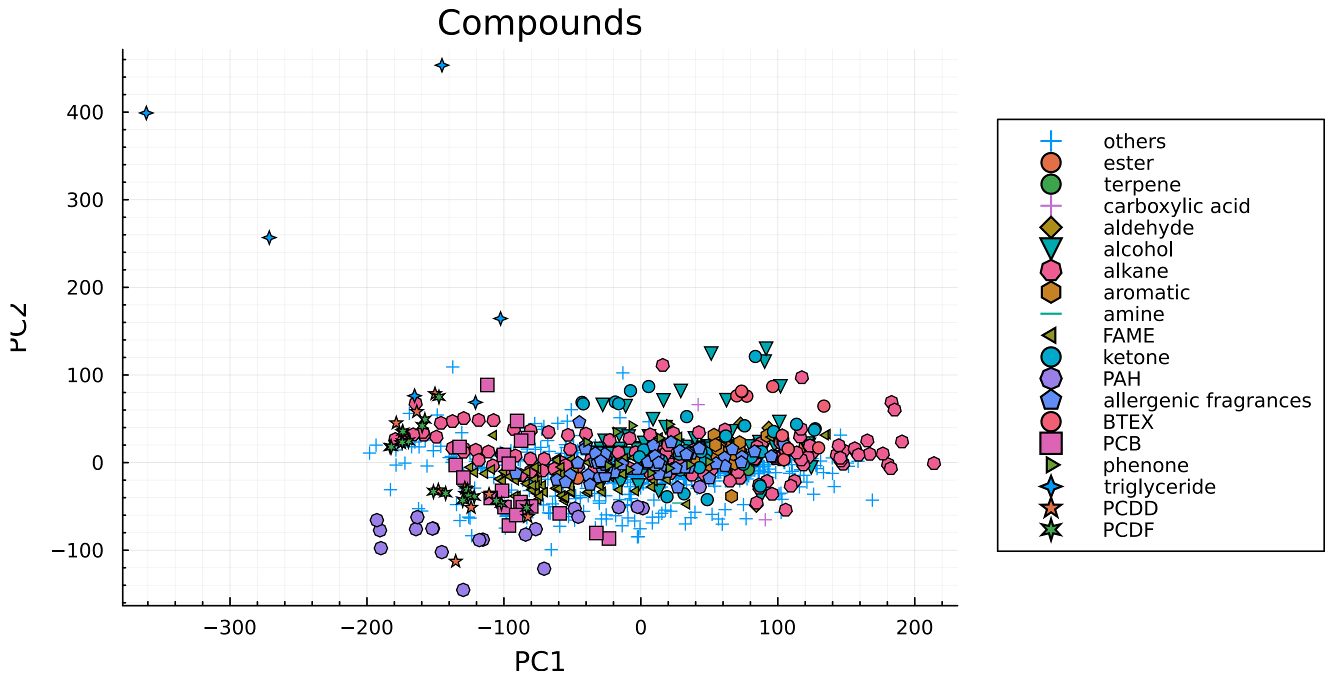


```
begin
    gr()
    KcentricPCA_Plot=Array{Any}(undef, size(SubstanceFilter)[1])
        KcentricPCA_Plot=Plots.scatter(xlabel="PC1", ylabel="PC2")
    for i=1:size(SubstanceFilter)[1]
                if i==1
        Name="others"
        else
        Name=SubstanceFilter[i].Cat_1[1]
        end
        Predict=predict(PCA_kcentric,Matrix(SubstanceFilter[i][!,index_Kcentric])')
        Plots.scatter!(KcentricPCA_Plot, Predict[1,:],Predict[2,:], label=Name,
        legend=:outerright, markers=Plots.supported_markers()[i],
        title="Compounds",dpi=500, size=(800,400), minorgrid=true)
    end
#ylims!(-300,0)
#xlims!(-500,200)
KcentricPCA_Plot
end
```

Skipped marker arg pixel.

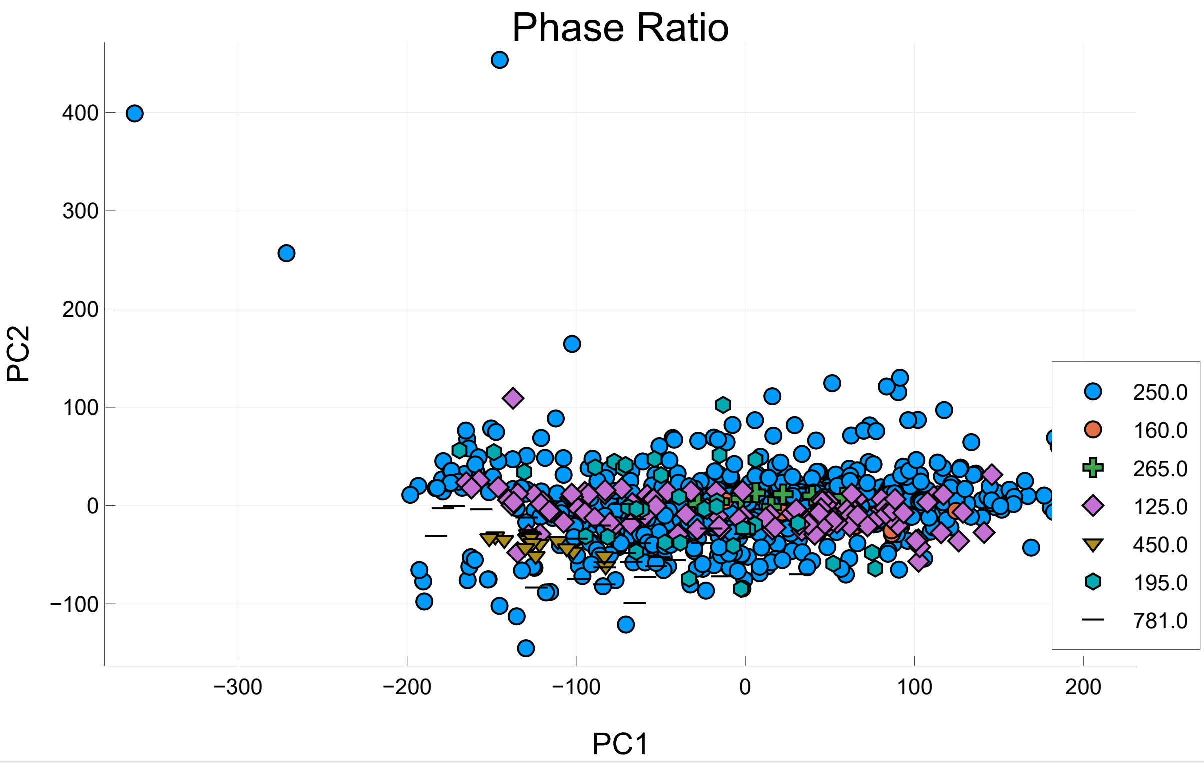


```
begin
plotly()
    KcentricPCA_Plot_beta=Array{Any}(undef, size(PhiFilter)[1])
        KcentricPCA_Plot_beta=Plots.scatter(xlabel="PC1", ylabel="PC2")
    for i=1:size(PhiFilter)[1]
        Predict=predict(PCA_kcentric,Matrix(PhiFilter[i][!,index_Kcentric])')
        Plots.scatter!(KcentricPCA_Plot_beta, Predict[1,:],Predict[2,:],
        label=PhiFilter[i].beta0[1], legend=:outerright,
        markers=Plots.supported_markers()[i], xlims=(), title="Phase Ratio")
    end
KcentricPCA_Plot_beta
end
```

```
Invalid limits for x axis. Limits should be a symbol, or a two-element tuple or
vector of numbers.
xlims = ()

Invalid limits for x axis. Limits should be a symbol, or a two-element tuple or
vector of numbers.
xlims = ()

Invalid limits for x axis. Limits should be a symbol, or a two-element tuple or
vector of numbers.
xlims = ()
```

```
2×394 Matrix{Float64}:
 -Inf  -Inf  -4984.9   -5566.26  -5982.55  …  -11552.5   -11671.9   -9750.02
 -Inf  -Inf  -1880.03  -2087.75  -2236.08      -4221.03   -4223.87  -3574.42
```

```
predict(PCA_kcentric,Matrix(SubstanceFilter[1][!,7:9])')
```

END

```
add_homologous_to_Cat! (generic function with 1 method)
```

```
filter_Cat (generic function with 1 method)
```

```
SubstFilter (generic function with 1 method)
```

```
SourceFilter (generic function with 1 method)
```

```
PaperPlot (generic function with 1 method)
```
